# Assessing the Permeability of Landscape Features to Animal Movement: Using Genetic Structure to Infer Functional Connectivity

**DOI:** 10.1371/journal.pone.0117500

**Published:** 2015-02-26

**Authors:** Sara J. Anderson, Elizabeth M. Kierepka, Robert K. Swihart, Emily K. Latch, Olin E. Rhodes

**Affiliations:** 1 Biosciences Department, Minnesota State University Moorhead, 1104 7^th^ Ave, Moorhead, MN, 56563, United States of America; 2 Department of Forestry and Natural Resources, 715 W. State Street, Purdue University, West Lafayette, IN, 47907, United States of America; 3 Behavioral and Molecular Ecology Group, Department of Biological Sciences, University of Wisconsin-Milwaukee, 3209 N. Maryland Ave., Milwaukee, WI, 53024, United States of America; 4 Savannah River Ecology Laboratory, PO Drawer E, Aiken, SC, 29802, United States of America; University of KwaZulu-Natal, SOUTH AFRICA

## Abstract

Human-altered environments often challenge native species with a complex spatial distribution of resources. Hostile landscape features can inhibit animal movement (i.e., genetic exchange), while other landscape attributes facilitate gene flow. The genetic attributes of organisms inhabiting such complex environments can reveal the legacy of their movements through the landscape. Thus, by evaluating landscape attributes within the context of genetic connectivity of organisms within the landscape, we can elucidate how a species has coped with the enhanced complexity of human altered environments. In this research, we utilized genetic data from eastern chipmunks (*Tamias striatus*) in conjunction with spatially explicit habitat attribute data to evaluate the realized permeability of various landscape elements in a fragmented agricultural ecosystem. To accomplish this we 1) used logistic regression to evaluate whether land cover attributes were most often associated with the matrix between or habitat within genetically identified populations across the landscape, and 2) utilized spatially explicit habitat attribute data to predict genetically-derived Bayesian probabilities of population membership of individual chipmunks in an agricultural ecosystem. Consistency between the results of the two approaches with regard to facilitators and inhibitors of gene flow in the landscape indicate that this is a promising new way to utilize both landscape and genetic data to gain a deeper understanding of human-altered ecosystems.

## Introduction

Fragmentation of natural landscapes by anthropogenic attributes, such as agriculture, urbanization, and transportation infrastructure, alters the manner in which animals utilize remnant native habitats [1], [2]. In fragmented agricultural ecosystems, native habitats are generally reduced in area, resulting in a reconfiguration and loss of connectivity between native habitats. The formerly continuous habitats may then be reduced to small patches separated by potentially inhospitable matrix habitat [3], [4]. As a result of the redistribution and loss of connectivity of habitat elements, successful movements of individuals between patches can be markedly reduced [5], either by direct mortality or reluctance to leave the safety of a known environment [6]. Over time, wildlife populations in fragmented landscapes can become genetically differentiated and may lose genetic diversity due to isolation and the acceleration of genetic drift [7–9].

Of particular concern in fragmented ecosystems is the concept that a long-term reduction in genetic diversity and impediments to gene flow can inhibit future adaptation of species [10]. However, if matrix habitats do not completely impede movement between patches, species inhabiting fragmented landscapes may function as a metapopulation. Metapopulations are typified by low levels of dispersal between populations and patterns of population extirpation and recolonization wherein populations inhabiting small, isolated patches are at greater risk of extinction than those inhabiting larger or more connected patches [11]. To help understand how to maintain connectivity between isolated patches, one method is to identify habitats that impede dispersal, a process that generally involves detecting barriers to gene flow.

In fragmented ecosystems, the term ‘barrier’ refers to possible impediments to individual movement that exist in the matrix between patches of ideal habitat. When matrix habitats prevent enough movement to cause genetic differentiation between patches, resultant patterns of genetic variation across a landscape should be correlated with matrix habitats. The desire to understand the relationship between landscape heterogeneity and genetic differentiation has largely led to the formation and prolific growth of the field of landscape genetics [12], [13]. The attraction of landscape genetic approaches for fragmented ecosystems is that assumptions about biological processes are more realistic than traditional population genetic models [14]. Importantly, landscape genetic models do not require *a priori* groupings and genetic patterns can be analyzed at the individual scale. Another benefit of using landscape genetics for fragmentation studies is its ability to disentangle multiple influences on gene flow because genetic variation is often influenced by multiple factors that operate at different spatial [15–18] and temporal scales [19], [20].

Landscape genetic models often depend on accurate definition of the functional biological extent of populations, particularly in management and conservation efforts [21], [22]. Unfortunately, defining biologically meaningful populations is especially problematic within the context of fragmented environments [23]. For example, understanding how factors like the spatial distribution of habitat attributes and the composition of the intervening matrix habitats contribute to the spatial extent of population structure is rarely intuitive. Fortunately, Bayesian approaches provide a method to use individual-based data for detection of underlying population structure at various spatial scales. Bayesian clustering algorithms have proven to be particularly useful for identifying major barriers to gene flow in a variety of species, often confirming expectations that topographic (e.g., [24–26]) and anthropogenic barriers (e.g., [27], [28]) limit the ability of animals to move freely through their environments. While these approaches have been extensively used to elucidate population subdivision resulting from prominent habitat features, Bayesian algorithms can also aid in evaluating how more cryptic factors impact gene flow in heterogeneous landscapes [29–31].

With sufficiently dense, spatially explicit genetic and landscape attribute data, we should be able to use Bayesian clustering tools to not only hypothesize about the presence of cryptic barriers to gene flow, but also to explicitly identify those habitat attributes contributing most significantly to landscape level permeability, even in complex landscapes with relatively subtle changes in fine-scale habitat attributes. One method to evaluate the effects of fragmentation is to utilize individual-based estimates of ancestry or assignment relating to each genetic cluster (i.e., *q*-values or posterior probabilities) inferred from Bayesian algorithms within landscape genetic models. These genetic variables can act as response variables in statistics that evaluate how landscape parameters influence genetic variation. For example, several studies have utilized genetic surfaces based on ancestry coefficients from Bayesian clustering programs to investigate how specific landscape features (i.e., unsuitable habitats, roads, and topographic barriers) impacted gene flow [18], [32]. Spatially explicit Bayesian algorithms (e.g., Geneland, [33], [34]) may be particularly suited to identifying the effects of fragmentation because they include spatial coordinates as priors, and thus, can infer spatially explicit boundary areas and, by extension, spatial isolating features between biologically relevant populations in the landscape (e.g., [27], [35], [36]).

The main goal of this study, therefore, was to assess the utility of spatially explicit Bayesian methods for detection of fine-scale habitat attributes that inhibit gene flow, leading to evidence of population subdivision, in cryptic, fragmented environments. To accomplish this objective we focused our research in the highly fragmented, human-dominated, agricultural landscape of northern Indiana. Our study species within this landscape was the eastern chipmunk (*Tamias striatus*), a species that exhibits substantial evidence of fine-scale genetic structure within our study area and for which a suite of 12 highly polymorphic microsatellites has been developed [37]. Chipmunks generally select forested habitats or those with adequate tree cover, so movement (and gene flow) was expected to be facilitated by forest and impeded by unsuitable habitats such as agriculture and roads. This study utilized spatially explicit Bayesian clustering to derive population structure from genotypic and spatial coordinate data for 1,422 eastern chipmunks distributed across 33 distinct 23 km^2^ study cells in northern Indiana to address two main objectives. First, we examined if the intervening habitat between pairs of patches with and without gene flow were characterized by different fine-scale habitat attributes. Second, we analyzed whether fine-scale land cover features could predict the probability of population membership calculated via spatially explicit Bayesian clustering methods.

## Methods

### Study Area and Sampling

**Fig 1:**
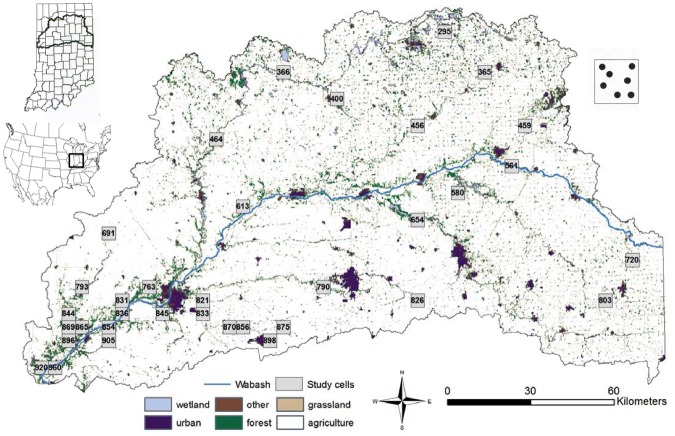
Map of study area in northern Indiana showing distribution of study cells. The blue line corresponds to the Wabash River, and land cover is colored. Right inset demonstrates several sample sites within each study cell. Left inset shows the study area location in North America and Indiana with counties outlined. All study cells are labeled by their ID.

Our study area was located in north-central Indiana, within the upper Wabash River basin (UWB; Fig. 1). The UWB drains over 20% (>20,000 km^2^) of the state [38] and contains eight major watersheds. Extensive land use change has occurred since the arrival of European settlers in the 17th century, primarily from forest to agriculture. Prior to European settlement, forest cover statewide is estimated to have been 87% [38], [39]. In contrast, forest cover during sample collection in UWB was about 8% (Fig. 1), compared to 19% statewide. Remaining native forests (predominantly oak-hickory-maple [*Quercus-Carya-Acer*]) in the basin are highly fragmented with the largest tracts confined to major drainages where floodplains or locally steep topography prohibits agriculture [38]. Possible corridors are dispersed throughout the region in various forms, such as small woodlots, fence rows, streams, shrubland, and windbreaks. The majority of land area was privately owned (96%) with 88% of the area designated as agriculture.

### Sample Collection

Eastern chipmunks were trapped at multiple sampling sites within 5, 18, and 12 of the 35, 23km^2^ study cells from late May to early August in 2001, 2002, 2003, respectively. Prior to each field season, all 30 X 30 m pixels in each cell were classified into 1 of 5 land cover categories (agriculture, forest, grassland, wetland, or urban), and then sampled according to a stratified-random design. To capture the variation within the study area, trapping grids were placed in sampling sites based on land cover so that natural land cover types (i.e., grassland, forest, and wetland; 27.8% of grids each) were disproportionally represented as compared to urban and agriculture (13.9 and 2.8% of all grids respectively). We randomly chose sampling sites (i.e., where trapping grids were placed) within patches of habitat by randomly selecting pixels of an appropriate habitat according to the predefined proportions stated above within each study cell (n = 35). Forest site selection had an additional step because they were also stratified according to forest patch size (small < 5 ha; medium 5–50 ha; large > 50 ha), and then were selected according to their log_10_ area within the three size categories. In total, a maximum of 45 sampling sites were selected per study cell in a summer [38], [40], and each forest habitat patch generally had 1–3 sampling sites [38]. A full description of study cell and sampling site selection is described in [38].

Each sampling site within a cell contained a grid of Fitch live traps (2001), Sherman live traps (2003), or a mixture of both (2002) spaced 15 m apart. Grid dimensions varied by trap year and patch size. Grids in 2001 were primarily 3 traps x 3 traps, with a few 7x7 grids where forest patch sizes were large enough to accommodate this arrangement. In 2002 and 2003, 5x5 grids were used whenever space allowed; otherwise 4x4 or 3x3 grids were used. If a forest patch was not large enough for a 3x3 grid, it was not sampled. Habitat corridors were identified as treed and non-treed land cover features less than 30 m wide and were fitted with 5x2 grids. Simultaneous trapping for raccoons was conducted at the edges of the trap grids to limit disturbance by raccoons to the small mammal traps.

Traps were baited with black-oil sunflower seeds. A pre-bait period with traps locked open occurred for 3 days, followed by a 5-day trap-check session, during which traps were checked twice daily. Each site had one trapping session. All animals were handled according to procedures approved by the Purdue Animal Care and Use Committee under protocol #01–024. Ear clips were taken from each individual using sterile scissors, and treated with ferric subsulfate in cases of excess bleeding. Animals were then released, and all tissues were stored in at -80°C prior to DNA extraction.

### DNA Extraction and Genotyping

We extracted DNA from ear tissue using an ammonium acetate protein precipitation protocol (modified form the PUREGENE kit; Gentra Systems) followed by an ethanol wash. Genomic DNA was run on 2% agarose gels stained with ethidium bromide to determine quality and quantity of DNA samples prior to genotyping. Amplification of twelve microsatellite loci [37] by multiplex PCR took place in 10 uL reactions volumes with 20 ng of template DNA, 0.2 mM of each dNTP, 1 U of *Taq* DNA polymerase (NEB) and 2X Thermopol reaction buffer (20 mM Tris-HCl, 10 mM (NH_4_)_2_SO_4_, 10 mM KCl, 2 mM MgSO_4_, 0.1% Triton X-100; NEB). Primer concentrations were adjusted so that intensities of the final products per multiplex reaction were approximately equal [37]. The amplification conditions included an initial denaturation step at 94°C for 2 min, then 35 cycles of 94°C for 30 s, annealing temperature [37] for 30 s, 72°C for 30 s, then a final extension of 72°C for 10 min and a soak at 60°C for 45 min. The PCR products were sized on an Applied Biosystems 3730 automated sequencer, and the genotypes were determined for all loci in all individuals using the software GeneMapper 3.7 (Applied Biosystems).

Several quality control measures were used to confirm the accuracy of genotypes. A negative control, two pre-amplified positive controls, and a concurrently amplified positive control were run on every 96-well plate. To standardize allele calling, ninety-two samples from each multiplex set were amplified twice, scored independently by two individuals, and examined for inconsistencies. Additionally, any ambiguous samples or samples with low quality flags according to GeneMapper 3.7 were re-amplified and genotyped again at all loci. Any individuals missing one or more genotypes were re-amplified in the multiplex reaction up to two times (the last using undiluted genomic DNA) in an attempt to obtain the genotype, which has the added benefit of confirming previous genotypes at the other loci in the multiplex. If there were still missing genotypes after re-amplifying the multiplex, we used single locus reactions to attempt to retrieve the missing genotypes.

Prior to statistical analyses, we used Cervus 2.0 [41] to calculate null allele frequencies as well as to identify and remove duplicate multilocus genotypes from the dataset. We tested for linkage disequilibrium and Hardy-Weinberg disequilibrium in Genepop 3.4 [42], [43]. To obtain standard errors <0.01, we increased default parameters to the following: 10,000 dememorization iterations, 2,000 batches, and 10,000 iterations per batch. F_IS_ values and 95% confidence intervals were calculated after 1000 permutations in R [44] using the package diveRsity (function “divBasic”; [45]). Finally, we tested for isolation-by-distance (IBD) across the entire study area using a simple Mantel test in the R package vegan [46] via the “mantel” function.

### Statistical Analysis

To investigate the influence of landscape attributes on the creation of genetic structure in eastern chipmunks, we utilized a hierarchical set of analyses involving five key steps. Each step is described in detail in the following sections. Briefly these were: 1) Conduct genetic-based estimation of populations to delineate population boundaries; 2) Define segments within and between populations to represent potential movement pathways of individuals; 3) Measure the proportion of each land cover type and the average probability of population membership in a series of segment widths to determine the appropriate sampling bandwidth for this system; 4) Utilize regression models to evaluate the role of fine-scale habitat attributes in genetic structure and the influence of landscape features on probability of population membership; and 5) Conduct model validation to assess whether the regression models are effective in predicting the outcomes for a test dataset.

We obtained genetic clusters (hereafter referred to as populations) using the Geneland package [33], [34] in R to cluster individuals within each of 33 of the 23 km^2^ study cells sampled for eastern chipmunks (two of the 35 study cells had too few samples to be analyzed). Genetic clustering within each study cell was based on multi-locus genotypes with spatial coordinates for all eastern chipmunks captured across all sample sites located within that particular study cell. Bayesian inference is used to estimate the number of populations under the assumption that spatial dependence exists among individuals, so that as geographical distance increases between two individuals, the joint probability that they belong to the same population decreases [34]. The method first estimates the number of populations (*k*), and then calculates assigning the probability of membership of individuals to each of *k* populations.

In initial runs of Geneland, we allowed the number of populations within each study cell to vary from 1–6 over 200,000 iterations (thinning = 40; 5,000 iterations retained) of the Markov chain. The number of polygons in the Voronoi tesselation was set close to the number of individuals, as suggested by the authors [34]. Allele frequencies were allowed to be correlated among populations, and an uncertainty radius of 100 m was included on spatial coordinates. The number of populations was estimated by the mode of the posterior distribution of *k*. Five independent runs were performed to ensure consistency in the estimation of *k*. If there were inconsistencies between runs (e.g., the modal *k* or individual assignments were different), we added additional runs with 400,000 iterations (thinning = 80; 5,000 iterations retained). Finally, Geneland may produce empty populations in addition to occupied populations, a phenomenon known as ‘ghost populations’ [34]. The authors suggest ignoring these empty populations and estimating *k* only from occupied populations, so we adjusted the estimated *k* accordingly.

After *k* was estimated, Geneland was run with a fixed *k* to obtain individual probabilities of population membership for each individual. The same parameters as when estimating *k* were used, except the number of iterations was increased to 400,000 (thinning = 80; 5,000 iterations retained). A grid of 100x100 pixels bound by the outer sample points was created, and the posterior probability of population membership was calculated for each pixel. This process creates a grid of posterior probabilities for each study cell, so study cells with strong genetic structure will have pixels with high posterior probabilities. We used FSTAT 2.9.3 [47] to calculate significance (1,200 randomizations) of pairwise F_ST_ values among populations of eastern chipmunks identified within each 23 km^2^ study cell as well as between study cells to confirm that the populations exhibited significant genetic differentiation.

**Fig 2:**
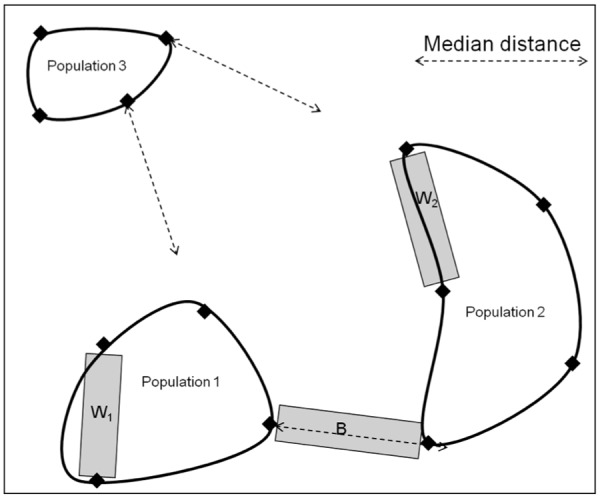
Diagram of point selection for habitat segments between and within genetically identified populations. The dashed line indicates the median distance between sample points within a population, averaged across all 70 populations in the study. In this example, the minimum distance between sample points from populations 1 and 2 is less than the median distance and qualified for analysis. However, the minimum distance between sample points from population 3 and the others exceed the median distance, so these did not qualify for a “B” segment. The shaded rectangle represents the segments, B = between populations and W = within populations.

To classify habitats within each 23 km^2^ study cell relative to permeability for gene flow, we first measured pairwise Euclidean distances between all sample sites contained within each population identified by Geneland, excluding those populations comprised of individuals sampled at only one sample point (i.e., no within population distance). Within each study cell, we used distance-based rules to classify line segments connecting sample point as between-population habitat (defined as occurring between two clearly identified populations) versus within-population habitat (defined as occurring within a clearly defined population). If two sample sites belonging to adjacent populations were geographically closer than the median distance of sample sites within those populations, clustering was assumed to be due to a barrier other than simple geographic distance. Pairs of sample sites meeting these criteria were classified as “between” populations, hereafter “B segments”. Alternatively, pairs of sample sites within each of the clearly identified populations on either side of the matrix that most closely matched the between-population distance were classified as “within” population, hereafter “W segments”. To avoid problems arising from spatial autocorrelation associated with the sampling of multiple W segments within any given population, we limited the total number of representative W segment habitats to 1 from each population joined by a B segment (see Fig. 2 for a diagram of this process). Populations that contained only one sample site could not produce W segments; however, this type of population could form one end of a B segment.

Due to the spatial distribution of sample sites within study cells, several (31%) populations were excluded as being associated with clearly defined B segments because the distance between those populations and neighboring populations was greater than the median distance between sample sites within populations. For these populations, the overall average B segment length (across all sample cells) was calculated (mean = 776 m, median = 760 m) and used to classify 1 W segment within each of these populations if possible. The only populations from which it was not possible to build a W segment were those containing one sample site.

For all segments (B and W), we calculated the proportional area of each of nine habitat types within each segment for a series of segment widths. The widths were varied to create a set of models from which we assessed the sampling bandwidth that best reflected the genetic probabilities of population membership associated with the habitat data. The widths we used were: 3 m, 10 m, 25 m, 50 m, 100 m, 200 m, 400 m, and 1,000 m. The 3 m segment width represents a straight line of minimal width between sample sites. The maximum width of 1,000 m represents a width beyond the maximum movement likely for an eastern chipmunk if an individual traveled along the edge of the defined segment. For each width examined, we recorded the area of each land cover type and the distance between the two sample sites defining the matrix using ArcGIS 9.2 (ESRI). For each segment, we also calculated the average probability of population membership, generated by Geneland, averaging across values for all pixels contained within the segment at each given width.

All defined segments were used to build a series of regression models designed to 1) identify differences in habitat characteristics that might exist between B and W segments of varying widths, and 2) to predict the average probability of population membership (genetic based) from habitat attributes. Eight fine-scale land cover types (forest, non-treed corridors, roads, grassland, shrubs, treed-corridors, urban, and water) served as predictor variables within our regression models. Non-treed corridors were comprised of a mix of grasses, shrubs, and some trees, but mature trees were not the dominant vegetative cover. The water variable included open water bodies as well as wetland areas. We excluded proportion of agriculture from all models due to its high negative correlation with proportion of forest cover. Correlation among the other habitat variables ranged widely across segment widths (S1 Table). Following calculation of land cover variables, we bootstrap resampled (R function “sample”) our full dataset (B and W segments and their associated land cover variables) 1000 times to calculate confidence intervals around all beta estimates as well as evaluate stochasticity within estimations.

To evaluate which land cover types characterized intervening habitats within or between populations, we used a set of logistic regression models to identify habitat characteristics that differed between B and W segments. The response variable was segment category (between genetically defined populations = 0, within genetically defined populations = 1). Stepwise model selection by AIC was used using the function “stepAIC” in the R package MASS [48]. Stepwise model selection calculates AIC for the full model, and the change in AIC for exclusion of each variable is calculated. If excluding a variable reduces AIC, the next model is evaluated with the remaining subset of variables. Previously excluded variables may be added back to the model if they lower AIC in a later reduced model. The final model is found when addition or exclusion of any variable does not improve the AIC of the model. The final models for each segment width were tested against the null using Chi-Squared tests. Since the same number of B and W segments was used across widths, the null deviance was equal among models as evaluated in [49]. We compared residual deviance among segment widths (i.e., highest residual deviance has the best fit) to determine the best sampling bandwidth for this system.

For our second objective, we used multiple linear regression models with the eight land cover variables as predictor variables to evaluate if land cover attributes can predict the probabilities of population membership calculated from Geneland (both B and W combined). Like the logistic regressions, models were run separately on each bootstrap iteration and segment width (3, 10, 25, 50, 100, 200, 400, and 1000 m). The dependent variable was the average probability of cluster membership across all pixels within each segment at each given width. We used an Anderson-Darling test (function “ad.test”) in the R package nortest [50] to test for normality in our probability data. The Anderson-Darling tests indicated probability values were not normal (A = 2.992, p < 0.001), so they underwent an arcsine square root transformation to conform to normality. We used the same stepwise selection by AIC as the logistic regression models to determine the best fitting model for each segment width. Significance of the final models for each segment width was assessed (probability of obtaining F-statistic greater than observed for the given degrees of freedom), and adjusted R^2^ values were used to assess the segment width that explained the largest proportion of the variance in probability of population membership. All regression calculations were performed in MASS.

To test the predictability of our regression models, we resampled 20% of the individuals within each original bootstrap iteration another 1000 times (total = 1,000,000 iterations per width). For the logistic regression models used to address our first objective, we calculated the probability of getting a B segment from the given composition of habitat and final logistic regression model coefficients for each segment width. A segment was predicted to belong to the W category if its probability was > 0.5, otherwise the category was B. We then compared the predicted result to the known category for each segment. To assess the strength of the multiple linear regression models for predicting probability of population membership, we calculated the predicted probabilities of population membership and 95% prediction intervals around each probability using the “predict.lm” function in MASS. If an observed probability fell within the 95% confidence intervals, the estimate was considered correct.

## Results

DNA from 1,448 eastern chipmunks was amplified at 12 microsatellite loci, which yielded a total of 17,140 of 17,376 (98.6%) possible genotypes. After duplicate genotypes from possible recaptures were removed, 1,422 chipmunks remained in the dataset. Test for Hardy-Weinberg and linkage disequilibrium did not indicate any consistent, significant deviations from expected values within or across loci, and null allele frequencies were generally low (0.022–0.123). All diversity metrics (observed and expected heterozygosities, numbers of alleles observed, allelic richness by rarefaction, and F_IS_ values) were similar between study cells (1-way ANOVA: F_Allelic richness_ = 1.112, F_observed heterozygosity_ = 1.023; all p ≥ 0.065; S2 Table). A simple Mantel test revealed significant IBD across the entire study area (r = 0.208, p = 0.003: S1 Fig.), and study cells exhibited significant genetic differentiation (F_ST_ range = 0.012–0.121).

In total, 70 populations were detected in 33 study cells, 8 cells had a single population and 26 had *k* ≥ 2 (S3 Table). All but one F_ST_ value between putative populations were significantly different from zero (S3 Table), supporting that clusters found in Geneland represent true population boundaries. The median distance between sample sites within populations was 1,389 m versus 760 m between populations. In 23 instances, the pairwise distance between sample sites within adjacent populations was less than the median within-population distance. Thus, 23 B segments were created for analyses using these pairs of sample sites. There were 47 instances where pairs of sample sites within each of the clearly identified populations closely matched the average between-population distance. We did not select multiple W segments for two reasons. First, including more W segments would likely introduce high amounts of spatial autocorrelation due to the strong IBD within our study landscapes. Second, we sought to prevent an overrepresentation of W segments within our analyses. In total, 47 W segments (1 W segment per population) and 23 B segments were utilized for our statistical analyses where each segment represented a single data point within our regression models.

Results from logistic regression model selection indicated that the proportions of forest, non-treed corridors, and grassland were consistent discriminatory variables for B or W segment habitats (Table 1). An increase in any of these variables increased the odds that the segment was within a population. Proportion of treed-corridors remained an important variable after model selection for 100 m, 200 m, and 400 m widths, and an increase in treed-corridors was associated with B segment habitats. At the matrix width of 1,000 m, only grassland was retained as an important variable. At this segment width, increases in the proportions of grasslands corresponded to W segment habitat. All beta estimates were highly consistent across the 1000 bootstrap iterations within each segment width as evidenced by the small standard errors and narrow 95% confidence intervals around each estimate. Percent deviance explained varied from 13.6–28.5% with 200 m (20.8% explained) and 400 m (28.5% explained) being the highest among the eight segment widths (Table 1).

**Table 1:** Final logistic regression models for habitat prediction of segment category in eastern chipmunks after stepwise selection from full models with response variable 0 = between population segment, 1 = within population segment.

Width	Variable	Estimate	Std. Error	Upper 95% CI	Lower 95% CI	Pr|t| > 0	Residual Deviance	p-value
3m							75.112	0.009
	Intercept	-1.206	0.006	-1.194	-1.218	0.136		
	Forest	2.580	0.005	2.589	2.571	0.014	% Exp	
	NTC	20.999	0.054	21.106	20.892	0.106	14.0	
	Grassland	8.501	0.027	8.554	8.447	0.089		
10m							74.186	0.020
	Intercept	-0.187	0.008	-0.171	-0.204	0.790		
	Forest	1.869	0.006	1.882	1.858	0.061	% Exp	
	Grassland	7.765	0.029	7.820	7.709	0.143	13.6	
	Shrubland	-5.878	0.018	-5.843	-5.912	0.177		
	Water	-6.431	0.022	-6.387	-6.475	0.102		
25m							72.298	0.006
	Intercept	-1.023	0.015	-0.994	-1.052	0.264		
	Forest	2.548	0.008	2.564	2.532	0.022	% Exp	
	NTC	22.487	0.065	22.614	22.360	0.110	17.3	
	Grassland	9.586	0.032	9.649	9.522	0.079		
50m							73.307	0.003
	Intercept	-1.491	0.004	-1.482	-1.499	0.053		
	Forest	2.816	0.005	2.825	2.807	0.009	% Exp	
	NTC	27.276	0.060	27.393	27.159	0.085	16.1	
	Grassland	9.970	0.037	10.043	9.898	0.062		
100m							68.377	0.002
	Intercept	-0.753	0.007	-0.739	-0.767	0.376		
	Forest	2.190	0.005	2.201	2.179	0.066	% Exp	
	NTC	26.597	0.099	26.792	26.402	0.136	21.8	
	Grassland	19.122	0.043	19.206	19.038	0.024		
	TC	-24.873	0.063	-24.749	-24.996	0.062		
200m							62.830	<0.001
	Intercept	-0.484	0.008	-0.469	-0.499	0.544		
	Forest	2.265	0.005	2.274	2.256	0.111	% Exp	
	NTC	39.698	0.112	39.918	39.478	0.072	28.1	
	Grassland	21.282	0.037	21.354	21.209	0.014		
	TC	-36.500	0.092	-36.349	-36.710	0.026		
	Shrubland	-7.515	0.021	-7.474	-7.555	0.218		
	Water	-7.015	0.049	-6.918	-7.112	0.119		
400m							62.455	<0.001
	Intercept	-0.520	0.006	-0.507	-0.532	0.541		
	Forest	2.701	0.005	2.711	2.690	0.085	% Exp	
	NTC	54.038	0.139	54.312	54.765	0.023	28.5	
	Grassland	18.304	0.044	18.390	18.218	0.019		
	TC	-53.261	0.105	-53.057	-53.467	0.010		
	Shrubland	-7.813	0.013	-7.788	-7.838	0.157		
	Water	-6.441	0.029	-6.385	-6.497	0.158		
1000m							74.491	0.007
	Intercept	-0.006	0.014	0.021	-0.033	0.467		
	Grassland	13.146	0.043	13.230	13.063	0.020	% Exp	
							14.8	

All metrics were calculated from the 1000 bootstrap iterations of each model, and parameters were included if stepwise AIC retained them in at least 900 bootstrap iterations. Shown for each segment width are: the selected variables, mean beta estimates, standard errors with 95% confidence intervals, and average p-values for each beta estimate (Pr|t| > 0). Statistics for the overall models include model residual deviance (null deviance = 87.350 for all models), average percent deviance explained by the model (% Exp), and p-value of the Chi-squared test of significance for the model. Land cover abbreviations are: NTC = non-treed corridor and TC = treed corridors.

Similar to the logistic regression analysis, the multiple linear regression retained forest, non-treed corridors, and grassland in the majority of best fit models (Table 2). The proportion of grassland within segments was retained in the final models identified for all eight segment widths and was positively related to probability of population membership in all models. Proportions of forest (3–100 m) and non-treed habitat (3–400m) also increased the probability of population membership and were retained in the majority of segment widths (Table 2). While proportion of water was retained in the majority of segment widths (10–200 m), it had a negative effect on probability of population membership. Standard errors around beta estimations were very small (0.001–0.822) across all widths and no 95% confidence intervals included zero, indicating all included variables impacted probability of membership. (The adjusted R^2^ value ranged from 0.074 to 0.206 (Table 2) with the highest values occurred at 200 and 400 m, and rapidly decreased at matrix widths 1000 m.

**Table 2:** Final multiple regression models for habitat composition prediction of probability of population membership for eastern chipmunks collected in northern Indiana after stepwise selection.

Width	Variable	Estimate	Std. Error	Upper 95% CI	Lower 95% CI	Pr|z| > 0	Adjusted R^2^	Model p-value
3m							0.150	0.028
	Intercept	0.627	0.001	0.629	0.626	<0.001		
	Forest	0.211	0.001	0.213	0.209	0.025		
	NTC	1.650	0.004	1.657	1.643	0.058		
	Grassland	0.812	0.001	0.814	0.809	0.117		
10m							0.127	0.013
	Intercept	0.923	0.001	0.924	0.922	<0.001		
	Forest	0.272	0.001	0.273	0.270	0.024		
	NTC	1.622	0.004	1.630	1.615	0.053		
	Grassland	0.819	0.822	0.817	0.002	0.119		
	Water	-0.840	0.002	-0.835	-0.844	0.086		
25m							0.143	0.009
	Intercept	0.929	0.001	0.931	0.927	<0.001		
	Forest	0.266	0.001	0.268	0.265	0.028		
	NTC	1.846	0.005	1.856	1.837	0.081		
	Grassland	0.837	0.001	0.840	0.834	0.048		
	Water	-0.876	0.003	-0.870	-0.883	0.077		
50m							0.158	0.005
	Intercept	0.943	0.001	0.944	0.942	<0.001		
	Forest	0.219	0.001	0.220	0.218	0.063		
	NTC	1.641	0.023	1.687	1.595	0.099		
	Grassland	1.018	0.001	1.021	1.015	0.012		
	Water	-0.944	0.002	-0.940	-0.948	0.053		
100m							0.147	0.007
	Intercept	0.919	0.001	0.920	0.918	<0.001		
	Forest	0.245	0.001	0.246	0.244	0.040		
	NTC	1.784	0.003	1.790	1.777	0.082		
	Grassland	0.930	0.001	0.932	0.928	0.011		
	Water	-0.758	0.002	-0.755	-0.761	0.109		
200m							0.186	0.004
	Intercept	1.070	0.001	1.072	1.068	<0.001		
	NTC	2.387	0.007	2.400	2.373	0.089		
	Grassland	1.375	0.002	1.379	1.371	0.002		
	TC	-2.793	0.007	-2.779	-2.807	0.027		
	Water	-0.789	0.002	-0.785	-0.793	0.097		
400m							0.206	0.001
	Intercept	1.058	0.001	1.059	1.057	<0.001		
	NTC	3.410	0.009	3.429	3.392	0.014		
	Grassland	1.418	0.002	1.421	1.415	0.001		
	TC	-4.391	0.006	-4.380	-4.402	0.002		
1000m							0.074	0.025
	Intercept	1.068	0.002	1.071	1.064	<0.001		
	Grassland	0.990	0.002	0.993	0.986	0.005		

All metrics were calculated via 1000 bootstrap iterations (average across 1000 iterations or 95% confidence intervals around beta estimates). Each segment width includes the selected variables, coefficient estimates, standard errors, 95% confidence intervals, p-values for each parameter, model adjusted R^2^, and p-value of the overall model. Variables that had an average p-value < 0.05 are given in bold. Note: NTC = non-treed corridors and TC = treed corridors.

**Fig 3:**
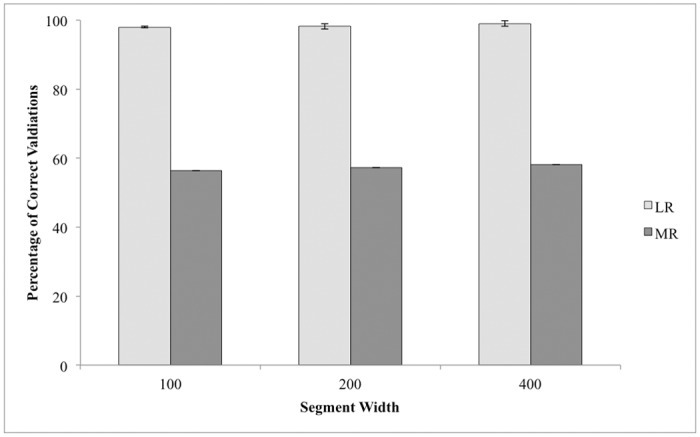
Proportion of correct validations for 100, 200, and 400m segment widths calculated based on 1,000,000 datasets. For logistic regressions (light grey), a correct validation occurred when a predicted value of a segment matched the classification of B (0) or W (1) in the observed dataset. A correct validation in the multiple regression (dark grey) was defined as when the observed probability of population membership for a segment fell within the 95% predicted intervals produced from the validation dataset. Logistic regressions were highly accurate with the majority of segments being correctly classified as B or W (98.01–99.02%) while multiple regressions had lower success (56.35–58.17%). Error bars correspond to 95% confidence intervals calculated across 14,000,0000 validated segments.

Collectively, our regressions suggest that the appropriate scale for assessing landscape permeability of small mammals like eastern chipmunks may be less than 1000 m in this landscape because best fit metrics (residual deviance and adjusted R^2^) peaked at 200–400 m. Therefore, validation was conducted on 100, 200, and 400 m for both logistic and multiple linear regressions (1,000,000 subsampled populations, 14,000,000 individuals total for each segment width). Assignment of the correct segment category (B or W) for each validated width occurred in 98.01–99.02% of the validation individuals within the logistic regression (14,000,000 individuals total; Fig. 3). In contrast, prediction intervals for 56.35–58.17% of individuals encompassed the observed values (Fig. 3). The drastic differences between the validations in logistic (98.01–99.02%) and multiple regression models (56.35–58.17%) likely stems from the definition of how a predicted value was deemed correct. Multiple regression prediction intervals were smaller than 0.5, the cut-off for the logistic regression, so the stringency in the 95% prediction intervals may explain the lower performance in the multiple regressions.

## Discussion

Although genetic drift as a consequence of isolation by distance likely contributes to variance among populations of eastern chipmunks in our study area, genetic differentiation clearly is not simply a function of spatial proximity in this landscape. We observed breaks in gene flow at distances of only a few hundred meters, well within the capabilities of chipmunks to traverse and less than the median distance between sample sites within populations. The results of our analyses indicate that probabilities of population membership identified across our study landscape likely reflect fine-scale physical and biological barriers to genetic exchange in this environment. Thus, landscape attributes that influence the successful movement, survival, and reproduction of individuals dictate the spatial distribution of populations across our study area, which is characterized by fine-scale habitat features representing pockets of habitat where gene flow readily occurs and gaps where gene flow is restricted. However, two sources of unexplained variance in our analyses that potentially could limit our ability to interpret the permeability of the various landscape attributes we examined are 1) the lack of data on other biological factors, such as predators and competitors, that may be associated with those attributes, and 2) lack of micro-habitat data (e.g., amount of woody debris, ground cover, or average DBH of trees per patch) which may affect the distribution of eastern chipmunks in the study area.

Results from the logistic and linear regression models indicated that forest, non-treed corridors, and grasslands are habitats that are contributing to gene flow and intrapopulation structure. Both the logistic and linear regression identified forest, non-treed corridors, and grasslands as important drivers of genetic structure across the majority of segment widths (3–400 m and 3–200 m in logistic and multiple regression respectively). We also observed little variation in the positive beta estimates in the 1000 bootstrap iterations, which provides robust evidence for the relationship between the land cover variables and whether segments were B or W. Overall, the consistency of variable selection by both statistical methods and precise beta estimates suggest that forest, non-treed corridors, and grasslands are important to gene flow in this agriculturally dominated landscape.

Forest, as the preferred habitat of eastern chipmunks, was expected to be highly associated with the probability of population membership. Therefore, if a high proportion of the land cover between two points was forest, the area was more likely to be within rather than between populations. Conversely, we expected grassland, a habitat type generally thought to be rather impermeable to small forest dwelling mammals due to its openness and relatively unprotected structure, to be negatively associated with gene flow. For example, [51] reported that eastern chipmunks rarely moved between forest and grassy fields, and [52] claimed that fencerows with only grassy vegetation were inhospitable to chipmunks. However, grassland was the only variable retained in models for all eight segment widths, with more grassland associated with habitat matrix within populations and increased probabilities of population membership. Although not an intuitive result, there is some evidence that eastern chipmunks prefer the forest-grassland ecotone to take advantage of a greater variety of food [53]. Another possible explanation for grassland promoting gene flow is some organisms move through inhospitable habitat quickly to reduce predation risk (e.g., [54–55]). Such compensatory movements [56] would predict that inhospitable habitats would not act barriers, and could explain why grasslands and non-treed corridors were found to promote gene flow.

Like grassland, non-treed corridors, a land cover variable thought to be unsuitable, also were identified as an important habitat attribute for predicting within population matrix and probability of population membership in both sets of models. A translocation study [57] demonstrated that chipmunks quickly left non-treed corridors after release and moved toward a forest patch, indicating an aversion to such habitat. Although habitat avoidance could suggest that dispersal does not occur in non-treed corridors, the mixture of grasses, shrubs, and trees within such areas may provide an abundance of resources for chipmunks. Thus, eastern chipmunks may find enough protection from predators and the elements in this habitat type to move freely between forest patches, even if individuals have no inclination to remain long-term in such habitat. Vertical stratification (herbs, shrubs, and trees) has been shown to have a positive influence on chipmunk abundance with shrub cover in particular being identified as a critical habitat element for maintenance of chipmunk populations [58]. Indeed, the importance of shrubs as a seasonal food source for chipmunks also has been noted [53], [59], even though dense, short vegetation may inhibit visual range [60].

In terms of the identification of habitat attributes that restrict gene flow of eastern chipmunks in this agricultural landscape, few variables stood out as clear barriers, although two variables of interest were treed corridors and water. Increases in the proportion of treed corridors increased the odds of a habitat being classified as between populations at 100–400m, and was statistically unimportant for predicting the probability of population membership except at 200 and 400 m. This result was very surprising as in this landscape we expected treed corridors to contribute similarly to genetic structure as did forests, since this land cover is simply narrow strips of forest. Instead, treed corridors seemed to function as a weak barrier to gene flow between populations in the handful of models in which it was selected. It is possible that a small number of eastern chipmunks could establish territories in these corridors and reside within them. Chipmunks are highly territorial, so these strips of treed corridors, if occupied, may act as barriers by stopping dispersing individuals and preventing them from reaching other populations. Additionally, predator species may be attracted to treed corridors and use these areas as concentrated feeding grounds, in which case this habitat type could become a population sink. Visual inspection of B segment areas revealed multiple instances of treed corridors occurring perpendicular to the segment between populations, often surrounded by agricultural fields. These perpendicular treed corridors may direct chipmunks to follow horizontally (with respect to the forest patches) for a time, and then turn back to the patch of origin instead of travelling on to a new patch. Alternatively, the association of treed corridors with large tracts of agriculture also may serve to falsely portray the role of treed corridors as isolating mechanisms by masking the underlying effect of agriculture. Further study of the role that treed corridors play in this landscape will be necessary to understand the biological basis for the observed relationship.

Interestingly, we did not detect an effect of roads on gene flow despite roads being implicated as barriers to dispersal for many species [61–64] including eastern chipmunks [62], [65], [66]. For example, [65] found that highways as wide as 90 m were as effective barriers to chipmunk movements as bodies of water twice as wide, and individual chipmunks seem to avoid the mowed roadside verge as well as the road surface regardless of traffic patterns [67]. However, road habitat was not selected as a variable in logistic or multiple regression models in any segment widths. Although we expected to detect a more pronounced effect of roads on gene flow, research also has demonstrated that chipmunks may be less averse to small dirt or gravel roads than to wider paved roads, some of which occur in our study areas [65]. Furthermore, barrier effects of roads combined with intrinsic population characteristics (e.g., high effective population sizes) may not yield sufficient genetic differentiation to constitute separate populations, even when field studies confirm reduced movement [68], [69]. Thus, while our analyses do not suggest that roads reduce gene flow and increase genetic differentiation between populations, more intensive sampling targeted at forest highway interfaces of differing types may be needed to clarify the specific effects of roads on gene flow in highly fragmented, agricultural landscapes.

One of the primary benefits of landscape genetics for studies of fragmentation is the use of the powerful analytical tools available to population geneticists to evaluate the complex nature of relationships between species ecology and landscape attributes. Bayesian algorithms are extensively used for population delineation in population and landscape genetics, but disentangling fine-scale processes can be difficult to interpret with a broad-scale analysis. In particular, the number of inferred clusters can be overestimated if subtle barriers [70] or isolation-by-distance [71] occur within a study area. Despite potential problems with inferring fine-scale processes via Bayesian clustering algorithms, our approach shows that they can be highly useful in fine-scale analyses even in the presence of confounding variables (e.g., high spatial autocorrelation). For example, outputs from Bayesian algorithms can be utilized to create genetic surfaces (e.g., [17], [32], [72], [73]), which can then further aid in population delineation as well as be used as genetic response variables within landscape genetic statistics (e.g., Mantel tests, regression, or constrained ordination). This study highlights that Bayesian programs can be utilized for many more applications beyond population assignment, and when paired with appropriate statistics like the regressions utilized in this study, can provide powerful evidence of how fragmentation impacts gene flow.

Another advantage of our approach was the ability to utilize regressions to evaluate both the influence of land cover variables on segment category type and the probability of population membership across the study area. Regression models provided the relative contribution of each habitat attribute as a barrier or facilitator of gene flow in the landscape (regression coefficients and measures of variance) and eliminated those that were unimportant for gene flow. Regression coefficients are particularly useful because unlike correlation coefficients in statistics like Mantel tests (see [74]), they can be used to predict how focal landscape variable influence genetic variation (as seen in the validation datasets). Furthermore, our approach suggested that the ideal sampling bandwidth for eastern chipmunks in this landscape was in segments approximately 400 m wide because performance (i.e., model fit statistics) occurred at 400 m wide for both logistic and multiple regression models. Landscape variables can have variable effects according to spatial scales (e.g., [15], [16], [75]), so our method provides a way to disentangle multiple influences on gene flow across spatial scales. Taken together, this approach is highly useful for informing management about how to maintain connectivity in fragmented landscapes because it can predict how landscape variables impact gene flow without *a priori* parameterization and explicitly incorporates spatial scale.

Fine-scale landscape genetic analyses such as the one undertaken in this study have great potential to contribute to conservation and corridor planning because they provide evidence how organisms move in heterogeneous landscapes. In particular, genetic data is often paired with individual-based movement analyses (e.g., [76–78]) that identify paths that organisms are most likely to travel based on costs assigned to different landscape features within a landscape. However, the accuracy of most individual-based modeling approaches rely heavily on the input cost values [79–81], which are generally derived as estimates provided by the investigator [81], with expert opinion and literature-derived information most often serving as the basis for assigning cost values [79], [81]. Several authors [81], [82] have argued that two main faults of individual-based modeling approaches is that we may never know the true cost of dispersal, and that cost values based on expert opinion may be based on the wrong type of data (e.g., presence/absence of habitat use rather than dispersal cost). If cost values are inaccurate for a given species, the resulting predictions of movement behaviors will be meaningless or misleading, the repercussions of which could lead to ineffective or detrimental conservation and management efforts. As such, the assignment of initial cost values is extremely important, and a pressing question for those who utilize all forms of individual-based modeling is how to most accurately assess cost values for habitats within a landscape.

The concept of using genetic information to inform individual-based analyses began to emerge due to concerns over improperly parameterized cost values. For example, recent studies have employed intense computational methods involving hundreds to thousands of least cost paths with different expert-derived cost values to reveal the parameter set that best matches a set of observed genetic parameters [82–84]. Also, methodologies such as [85] have recently been developed to help optimize and develop unbiased cost values for landscape genetic studies. Our approach represents another potential method to parameterize landscapes without including human bias because no *a priori* assignment of cost values is needed. Instead, regression coefficients derived from models developed in independent landscapes like our study could also be used to parameterize resistance surfaces. Once developed, genetically informed individual-based models could be used for a variety of purposes such as comparison to other parameterization (e.g., expert opinion, presence-absence) or optimization ([84], [85]) methods, mapping possible movement corridors, estimating how functional connectivity may change under future land use alterations, or predicting changes in ecological and genetic parameters associated with changing land use practices.

Elucidating the complex relationships between landscape features and gene flow continues to be important in our human-dominated ecosystems as conservation experts try to plan for future challenges by mitigating isolating factors and improving connectivity of natural populations. In this study, utilizing both spatially explicit genetic and landscape attribute data, we have been successful in identifying landscape elements that affect gene flow within and among eastern chipmunk populations inhabiting a highly fragmented agricultural ecosystem. The results of our analyses are intriguing, given that the estimated permeability of both grasslands and treed corridors to chipmunk movement were counterintuitive and thus, point to the need for further study of how this species actually utilizes these landscape attributes. Ultimately, the approaches outlined herein can be extended to a variety of species and the results utilized to more accurately parameterize individual-based models for application to a wide variety of conservation and management issues.

## Supporting Information

S1 Table Correlation coefficients and standard deviation of landscape features found in the segments between populations (below diagonal) and within populations (above diagonal) across the eight matrix widths. Abbreviations are as follows: ag = agriculture, ntc = non-treed corridors and tc = treed corridors.
(DOCX)

S2 Table Microsatellite locus information for eastern chipmunks sampled in northern Indiana during 2001–2003 by study cell. Sample size (N), number of alleles (Na), observed (Ho) and expected heterozygosity (He), allelic richness (A_R_), and F_IS_ with the upper and lower 95% confidence intervals are listed per locus and sample cell. Bold F_IS_ values indicate a significant deficiency of heterozygotes.
(DOCX)

S3 Table Number of genetic clusters estimated from Geneland (*k*) and F_ST_ for each study cell. Global F_ST_ values were calculated for each study cell as well as pair-wise F_ST_ values between all putative clusters within a study cell. All but one F_ST_ between putative clusters were significantly different from zero.
(DOCX)

S1 Fig Plot of Euclidean distance versus F_ST_/1—F_ST_ between 33 study cells included in this study. We recorded a positive relationship between geographic and genetic distance, consistent with IBD (r = 0.208, p = 0.003).
(TIF)
