# Supplementary material for: Assessing the Permeability of Landscape Features to Animal Movement: Using Genetic Structure to Infer Functional Connectivity
Source: PLoS One. 2015 Feb 26;10(2):e0117500. doi: 10.1371/journal.pone.0117500 (PMC4342345; doi:10.1371/journal.pone.0117500)
Supplement: S1 Table — Abbreviations are as follows: ag = agriculture, ntc = non-treed corridors and tc = treed corridors. (DOCX) [file pone.0117500.s001.docx]

**Table S1:** **Correlation coefficients and standard deviation of landscape features found in the segments between populations (below diagonal) and within populations (above diagonal) across the eight matrix widths.**

Abbreviations are as follows: ag = agriculture, ntc = non-treed corridors and tc = treed corridors.

|  | *%forest* | *%ntc* | *%road* | *%grass* | *%shrub* | *%ag* | *%tc* | *%urban* | *%wetland* |
| --- | --- | --- | --- | --- | --- | --- | --- | --- | --- |
| %forest | --- | -0.22 (0.07) | -0.17 (0.07) | -0.17 (0.19) | 0.02 (0.06) | -0.81 (0.01) | -0.45 (0.12) | -0.18 (0.09) | -0.02 (0.1) |
| %ntc | -0.35 (0.08) | --- | 0.25 (0.11) | -0.05 (0.05) | 0.09 (0.02) | 0.12 (0.07) | 0.13 (0.14) | -0.06 (0.04) | -0.09 (0.06) |
| %road | -0.22 (0.06) | 0.37 (0.16) | --- | -0.07 (0.05) | 0.06 (0.07) | 0.12 (0.11) | 0.11 (0.1) | 0.09 (0.09) | -0.08 (0.05) |
| %grass | -0.01 (0.04) | -0.05 (0.16) | 0.12 (0.22) | --- | 0.03 (0.1) | -0.28 (0.15) | 0.38 (0.2) | 0.22 (0.08) | 0.04 (0.04) |
| %shrub | -0.12 (0.03) | 0.01 (0.32) | 0.05 (0.32) | 0.20 (0.36) | --- | -0.18 (0.07) | 0.04 (0.13) | -0.03 (0.11) | -0.04 (0.12) |
| %ag | -0.77 (0.03) | 0.27 (0.14) | 0.12 (0.15) | -0.26 (0.1) | -0.38 (0.03) | --- | 0.12 (0.05) | -0.17 (0.09) | -0.17 (0.11) |
| %tc | -0.08 (0.07) | -0.01 (0.14) | 0.11 (0.15) | 0.66 (0.14) | 0.08 (0.16) | -0.15 (0.09) | --- | 0.14 (0.06) | 0.15 (0.23) |
| %urban | -0.24 (0.08) | 0.2 (0.05) | 0.23 (0.09) | 0.09 (0.09) | 0.03 (0.14) | 0.05 (0.13) | -0.11 (0.08) | --- | -0.05 (0.15) |
| %wetland | 0.28 (0.08) | -0.19 (0.06) | -0.29 (0.02) | -0.20 (0.03) | -0.14 (0.03) | -0.43 (0.08) | -0.08 (0.16) | -0.18 (0.09) | --- |
