# Supplementary material for: Assessing the Permeability of Landscape Features to Animal Movement: Using Genetic Structure to Infer Functional Connectivity
Source: PLoS One. 2015 Feb 26;10(2):e0117500. doi: 10.1371/journal.pone.0117500 (PMC4342345; doi:10.1371/journal.pone.0117500)
Supplement: S2 Table — Sample size (N), number of alleles (Na), observed (Ho) and expected heterozygosity (He), allelic richness (AR), and FIS with the upper and lower 95% confidence intervals are listed per locus and sample cell. Bold FIS values indicate a significant deficiency of heterozygotes. (DOCX) [file pone.0117500.s002.docx]

**Table S2: Microsatellite locus information for eastern chipmunks sampled in northern Indiana during 2001-2003 by study cell.**

Sample size (N), number of alleles (Na), observed (Ho) and expected heterozygosity (He), allelic richness (A_R_), and F_IS_ with the upper and lower 95% confidence intervals are listed per locus and sample cell. Bold F_IS_ values indicate a significant deficiency of heterozygotes.

| Cell | Locus | N | Na | Ho | He | A_R_ | F_IS_ | F_IS_ Upper | F_IS_ Lower |
| --- | --- | --- | --- | --- | --- | --- | --- | --- | --- |
| 295 | | |  |  |  |  |  |  |  |
|  | EACH01 | 144 | 7 | 0.63 | 0.65 | 4.72 | 0.034 | 0.134 | -0.005 |
|  | EACH02 | 143 | 10 | 0.69 | 0.77 | 6.78 | 0.098 | 0.193 | -0.001 |
|  | EACH03 | 144 | 9 | 0.66 | 0.73 | 5.46 | 0.099 | 0.201 | -0.003 |
|  | EACH04 | 144 | 16 | 0.83 | 0.89 | 9.68 | 0.062 | 0.134 | -0.005 |
|  | EACH05 | 143 | 9 | 0.80 | 0.82 | 6.46 | 0.025 | 0.108 | -0.063 |
|  | EACH06 | 143 | 19 | 0.80 | 0.87 | 10.14 | 0.071 | 0.146 | 0.006 |
|  | EACH07 | 144 | 14 | 0.73 | 0.80 | 7.54 | 0.086 | 0.179 | 0.000 |
|  | EACH08 | 144 | 8 | 0.66 | 0.73 | 5.39 | 0.101 | 0.200 | 0.009 |
|  | EACH09 | 144 | 17 | 0.83 | 0.88 | 9.93 | 0.058 | 0.125 | -0.008 |
|  | EACH10 | 143 | 7 | 0.48 | 0.54 | 4.75 | 0.105 | 0.214 | -0.001 |
|  | EACH11 | 144 | 14 | 0.75 | 0.85 | 7.68 | 0.114 | 0.195 | 0.037 |
|  | EACH12 | 143 | 11 | 0.55 | 0.61 | 6.12 | 0.095 | 0.196 | -0.002 |
|  | Overall | 144 | 11.75 | 0.70 | 0.76 | 7.05 | **0.076** | **0.1036** | **0.051** |
| 365 | |  |  |  |  |  |  |  |  |
|  | EACH01 | 30 | 8 | 0.67 | 0.70 | 5.63 | 0.054 | 0.280 | -0.150 |
|  | EACH02 | 31 | 9 | 0.61 | 0.79 | 6.80 | 0.220 | 0.432 | 0.026 |
|  | EACH03 | 31 | 8 | 0.65 | 0.69 | 4.84 | 0.071 | 0.302 | -0.149 |
|  | EACH04 | 31 | 9 | 0.84 | 0.83 | 7.83 | -0.008 | 0.130 | -0.144 |
|  | EACH05 | 31 | 7 | 0.71 | 0.73 | 5.90 | 0.029 | 0.227 | -0.157 |
|  | EACH06 | 31 | 6 | 0.77 | 0.78 | 5.46 | 0.011 | 0.206 | -0.167 |
|  | EACH07 | 31 | 11 | 0.87 | 0.87 | 8.75 | -0.005 | 0.142 | -0.127 |
|  | EACH08 | 31 | 4 | 0.65 | 0.73 | 3.98 | 0.116 | 0.326 | -0.100 |
|  | EACH09 | 31 | 12 | 0.97 | 0.86 | 8.50 | -0.131 | -0.032 | -0.206 |
|  | EACH10 | 31 | 7 | 0.48 | 0.56 | 5.09 | 0.136 | 0.375 | -0.082 |
|  | EACH11 | 31 | 9 | 0.77 | 0.81 | 6.82 | 0.048 | 0.217 | -0.113 |
|  | EACH12 | 31 | 6 | 0.77 | 0.78 | 5.46 | 0.011 | 0.206 | -0.167 |
|  | Overall | 31 | 8.00 | 0.70 | 0.73 | 6.15 | 0.041 | 0.087 | -0.008 |
| 366 | |  |  |  |  |  |  |  |  |
|  | EACH01 | 134 | 8 | 0.73 | 0.72 | 5.78 | -0.013 | 0.078 | -0.094 |
|  | EACH02 | 135 | 8 | 0.76 | 0.79 | 6.49 | 0.047 | 0.136 | -0.036 |
|  | EACH03 | 135 | 10 | 0.74 | 0.77 | 6.86 | 0.037 | 0.120 | -0.041 |
|  | EACH04 | 135 | 14 | 0.87 | 0.88 | 9.34 | 0.003 | 0.066 | -0.054 |
|  | EACH05 | 134 | 10 | 0.85 | 0.81 | 6.97 | -0.052 | 0.025 | -0.120 |
|  | EACH06 | 134 | 14 | 0.64 | 0.82 | 8.56 | **0.213** | **0.305** | **0.122** |
|  | EACH07 | 133 | 14 | 0.62 | 0.74 | 7.30 | **0.162** | **0.259** | **0.061** |
|  | EACH08 | 133 | 7 | 0.66 | 0.67 | 4.48 | 0.015 | 0.130 | -0.100 |
|  | EACH09 | 134 | 16 | 0.75 | 0.84 | 9.28 | 0.116 | 0.201 | 0.043 |
|  | EACH10 | 132 | 6 | 0.41 | 0.41 | 4.10 | 0.012 | 0.126 | -0.098 |
|  | EACH11 | 135 | 13 | 0.80 | 0.82 | 7.54 | 0.024 | 0.100 | -0.049 |
|  | EACH12 | 133 | 11 | 0.73 | 0.73 | 8.10 | 0.005 | 0.092 | -0.078 |
|  | Overall | 135 | 10.92 | 0.71 | 0.75 | 7.07 | **0.049** | **0.075** | **0.025** |
| 400 | |  |  |  |  |  |  |  |  |
|  | EACH01 | 28 | 4 | 0.68 | 0.67 | 3.92 | -0.017 | 0.214 | -0.236 |
|  | EACH02 | 28 | 8 | 0.71 | 0.74 | 6.49 | 0.037 | 0.262 | -0.166 |
|  | EACH03 | 28 | 8 | 0.82 | 0.78 | 6.71 | -0.051 | 0.135 | -0.215 |
|  | EACH04 | 28 | 6 | 0.79 | 0.75 | 5.15 | -0.042 | 0.188 | -0.261 |
|  | EACH05 | 28 | 6 | 0.68 | 0.67 | 4.45 | -0.138 | 0.117 | -0.347 |
|  | EACH06 | 28 | 8 | 0.68 | 0.72 | 5.93 | 0.052 | 0.294 | -0.188 |
|  | EACH07 | 28 | 8 | 0.75 | 0.81 | 6.70 | 0.073 | 0.255 | -0.106 |
|  | EACH08 | 28 | 5 | 0.75 | 0.66 | 4.06 | -0.138 | 0.117 | -0.347 |
|  | EACH09 | 28 | 9 | 0.75 | 0.75 | 6.95 | -0.006 | 0.197 | -0.186 |
|  | EACH10 | 28 | 6 | 0.61 | 0.63 | 4.72 | 0.082 | 0.327 | -0.153 |
|  | EACH11 | 28 | 6 | 0.75 | 0.77 | 5.35 | 0.023 | 0.237 | -0.151 |
|  | EACH12 | 28 | 10 | 0.61 | 0.63 | 6.77 | 0.031 | 0.228 | -0.145 |
|  | Overall | 28 | 7.00 | 0.71 | 0.72 | 5.60 | 0.003 | 0.072 | -0.062 |
| 456 | |  |  |  |  |  |  |  |  |
|  | EACH01 | 52 | 8 | 0.67 | 0.76 | 5.72 | 0.112 | 0.262 | -0.054 |
|  | EACH02 | 53 | 10 | 0.85 | 0.82 | 7.15 | -0.035 | 0.080 | -0.133 |
|  | EACH03 | 53 | 9 | 0.79 | 0.76 | 6.19 | -0.040 | 0.114 | -0.180 |
|  | EACH04 | 52 | 11 | 0.87 | 0.86 | 8.30 | -0.008 | 0.101 | -0.106 |
|  | EACH05 | 53 | 8 | 0.77 | 0.75 | 5.52 | -0.030 | 0.121 | -0.174 |
|  | EACH06 | 51 | 11 | 0.88 | 0.87 | 8.17 | -0.019 | 0.094 | -0.114 |
|  | EACH07 | 52 | 10 | 0.81 | 0.81 | 7.28 | 0.009 | 0.136 | -0.097 |
|  | EACH08 | 52 | 6 | 0.60 | 0.64 | 4.72 | 0.071 | 0.241 | -0.088 |
|  | EACH09 | 52 | 11 | 0.85 | 0.82 | 7.96 | -0.028 | 0.095 | -0.152 |
|  | EACH10 | 52 | 4 | 0.42 | 0.46 | 3.51 | 0.089 | 0.266 | -0.103 |
|  | EACH11 | 52 | 13 | 0.81 | 0.85 | 9.13 | 0.052 | 0.167 | -0.055 |
|  | EACH12 | 52 | 8 | 0.71 | 0.74 | 6.24 | 0.042 | 0.183 | -0.093 |
|  | Overall | 53 | 9.08 | 0.75 | 0.76 | 6.66 | 0.014 | 0.058 | -0.030 |
| 580 | |  |  |  |  |  |  |  |  |
|  | EACH01 | 52 | 7 | 0.65 | 0.67 | 5.85 | 0.022 | 0.184 | -0.111 |
|  | EACH02 | 52 | 6 | 0.65 | 0.78 | 5.46 | 0.158 | 0.314 | 0.007 |
|  | EACH03 | 52 | 9 | 0.88 | 0.81 | 7.22 | -0.095 | -0.005 | -0.178 |
|  | EACH04 | 52 | 11 | 0.85 | 0.82 | 8.81 | -0.027 | 0.074 | -0.117 |
|  | EACH05 | 52 | 8 | 0.83 | 0.82 | 7.20 | -0.004 | 0.107 | -0.115 |
|  | EACH06 | 52 | 8 | 0.75 | 0.68 | 5.27 | -0.110 | 0.043 | -0.274 |
|  | EACH07 | 52 | 10 | 0.83 | 0.79 | 6.70 | -0.052 | 0.078 | -0.167 |
|  | EACH08 | 52 | 5 | 0.63 | 0.61 | 4.05 | -0.042 | 0.156 | -0.243 |
|  | EACH09 | 51 | 11 | 0.75 | 0.83 | 7.63 | 0.099 | 0.234 | -0.029 |
|  | EACH10 | 52 | 7 | 0.65 | 0.69 | 5.51 | 0.053 | 0.216 | -0.107 |
|  | EACH11 | 52 | 13 | 0.71 | 0.79 | 8.01 | 0.099 | 0.222 | -0.025 |
|  | EACH12 | 51 | 7 | 0.73 | 0.74 | 5.89 | 0.018 | 0.140 | -0.105 |
|  | Overall | 52 | 8.50 | 0.74 | 0.75 | 6.47 | 0.012 | 0.055 | -0.032 |
| 654 | |  |  |  |  |  |  |  |  |
|  | EACH01 | 67 | 6 | 0.84 | 0.78 | 5.17 | -0.077 | 0.041 | -0.184 |
|  | EACH02 | 67 | 6 | 0.75 | 0.74 | 4.63 | -0.009 | 0.143 | -0.152 |
|  | EACH03 | 67 | 7 | 0.82 | 0.77 | 5.88 | -0.070 | 0.045 | -0.176 |
|  | EACH04 | 67 | 12 | 0.78 | 0.84 | 8.30 | 0.076 | 0.186 | -0.027 |
|  | EACH05 | 67 | 9 | 0.78 | 0.76 | 6.16 | -0.016 | 0.110 | -0.135 |
|  | EACH06 | 67 | 11 | 0.84 | 0.77 | 7.15 | -0.079 | 0.032 | -0.180 |
|  | EACH07 | 67 | 13 | 0.61 | 0.86 | 8.37 | **0.287** | **0.423** | **0.165** |
|  | EACH08 | 67 | 7 | 0.69 | 0.72 | 4.97 | 0.048 | 0.187 | -0.082 |
|  | EACH09 | 67 | 17 | 0.82 | 0.89 | 10.96 | 0.079 | 0.181 | -0.015 |
|  | EACH10 | 66 | 8 | 0.47 | 0.52 | 5.18 | 0.101 | 0.293 | -0.071 |
|  | EACH11 | 67 | 12 | 0.88 | 0.82 | 8.65 | -0.072 | 0.021 | -0.164 |
|  | EACH12 | 66 | 7 | 0.50 | 0.51 | 4.54 | 0.012 | 0.173 | -0.126 |
|  | Overall | 67 | 9.58 | 0.73 | 0.75 | 6.66 | 0.025 | 0.061 | -0.008 |
| 459 | |  |  |  |  |  |  |  |  |
|  | EACH01 | 14 | 4 | 1.0 | 0.67 | 3.65 | -0.491 | -0.356 | -0.677 |
|  | EACH02 | 14 | 7 | 0.79 | 0.81 | 6.16 | 0.025 | 0.292 | -0.221 |
|  | EACH03 | 14 | 7 | 0.57 | 0.76 | 6.06 | 0.243 | 0.549 | -0.066 |
|  | EACH04 | 14 | 8 | 0.93 | 0.78 | 6.97 | -0.190 | -0.014 | -0.367 |
|  | EACH05 | 14 | 6 | 0.79 | 0.71 | 4.98 | -0.108 | 0.249 | -0.428 |
|  | EACH06 | 14 | 7 | 0.79 | 0.70 | 6.34 | -0.116 | 0.200 | -0.302 |
|  | EACH07 | 14 | 6 | 0.79 | 0.73 | 5.56 | -0.108 | 0.249 | -0.428 |
|  | EACH08 | 14 | 6 | 0.79 | 0.74 | 5.50 | -0.055 | 0.221 | -0.279 |
|  | EACH09 | 14 | 9 | 0.93 | 0.86 | 7.84 | -0.083 | 0.107 | -0.235 |
|  | EACH10 | 14 | 5 | 0.43 | 0.62 | 4.75 | 0.309 | 0.665 | 0.034 |
|  | EACH11 | 14 | 5 | 0.79 | 0.77 | 4.64 | -0.027 | 0.297 | -0.359 |
|  | EACH12 | 14 | 5 | 0.50 | 0.46 | 4.15 | -0.083 | 0.179 | -0.288 |
|  | Overall | 14 | 6.25 | 0.76 | 0.72 | 5.55 | -0.054 | 0.030 | -0.139 |
| 464 | |  |  |  |  |  |  |  |  |
|  | EACH01 | 158 | 5 | 0.57 | 0.66 | 4.34 | 0.137 | 0.243 | 0.033 |
|  | EACH02 | 163 | 10 | 0.72 | 0.76 | 6.45 | 0.057 | 0.140 | -0.024 |
|  | EACH03 | 147 | 7 | 0.66 | 0.69 | 5.24 | 0.042 | 0.137 | -0.047 |
|  | EACH04 | 152 | 11 | 0.87 | 0.85 | 7.73 | -0.022 | 0.043 | -0.078 |
|  | EACH05 | 163 | 9 | 0.71 | 0.78 | 6.31 | 0.096 | 0.184 | 0.012 |
|  | EACH06 | 161 | 13 | 0.74 | 0.76 | 8.04 | 0.027 | 0.096 | -0.041 |
|  | EACH07 | 161 | 15 | 0.69 | 0.85 | 8.81 | **0.195** | **0.277** | **0.119** |
|  | EACH08 | 162 | 4 | 0.53 | 0.58 | 3.43 | 0.076 | 0.198 | -0.044 |
|  | EACH09 | 156 | 14 | 0.88 | 0.88 | 9.64 | 0.004 | 0.063 | -0.049 |
|  | EACH10 | 145 | 4 | 0.50 | 0.45 | 3.22 | -0.116 | -0.023 | -0.201 |
|  | EACH11 | 156 | 13 | 0.77 | 0.77 | 6.70 | -0.006 | 0.077 | -0.085 |
|  | EACH12 | 151 | 13 | 0.73 | 0.74 | 7.97 | 0.013 | 0.087 | -0.057 |
|  | Overall | 163 | 9.83 | 0.70 | 0.73 | 6.49 | **0.046** | **0.073** | **0.021** |
| 865 | |  |  |  |  |  |  |  |  |
|  | EACH01 | 27 | 6 | 0.68 | 0.66 | 4.98 | -0.021 | 0.176 | -0.211 |
|  | EACH02 | 27 | 5 | 0.39 | 0.55 | 3.65 | 0.287 | 0.581 | -0.016 |
|  | EACH03 | 26 | 6 | 0.85 | 0.75 | 5.21 | -0.131 | 0.042 | -0.280 |
|  | EACH04 | 26 | 6 | 0.78 | 0.74 | 5.47 | -0.049 | 0.145 | -0.250 |
|  | EACH05 | 27 | 6 | 0.75 | 0.76 | 5.50 | 0.013 | 0.214 | -0.173 |
|  | EACH06 | 27 | 11 | 0.86 | 0.87 | 8.90 | 0.010 | 0.153 | -0.111 |
|  | EACH07 | 27 | 6 | 0.75 | 0.71 | 5.37 | -0.063 | 0.130 | -0.247 |
|  | EACH08 | 27 | 4 | 0.64 | 0.62 | 3.41 | -0.032 | 0.205 | -0.261 |
|  | EACH09 | 27 | 16 | 0.93 | 0.90 | 11.39 | -0.033 | 0.066 | -0.114 |
|  | EACH10 | 26 | 6 | 0.30 | 0.27 | 3.63 | -0.102 | -0.041 | -0.177 |
|  | EACH11 | 27 | 8 | 0.86 | 0.80 | 6.46 | -0.072 | 0.095 | -0.215 |
|  | EACH12 | 26 | 8 | 0.93 | 0.84 | 6.99 | -0.108 | 0.012 | -0.210 |
|  | Overall | 27 | 7.33 | 0.73 | 0.71 | 5.91 | -0.029 | 0.021 | -0.079 |
| 821 | |  |  |  |  |  |  |  |  |
|  | EACH01 | 19 | 5 | 0.72 | 0.69 | 4.52 | -0.045 | 0.216 | -0.271 |
|  | EACH02 | 19 | 5 | 0.61 | 0.64 | 4.58 | 0.050 | 0.351 | -0.239 |
|  | EACH03 | 19 | 7 | 0.83 | 0.77 | 5.97 | -0.089 | 0.090 | -0.239 |
|  | EACH04 | 19 | 7 | 0.83 | 0.81 | 6.33 | -0.027 | 0.172 | -0.227 |
|  | EACH05 | 19 | 4 | 0.83 | 0.65 | 3.54 | -0.280 | -0.072 | -0.475 |
|  | EACH06 | 18 | 7 | 0.88 | 0.81 | 6.04 | -0.085 | 0.137 | -0.255 |
|  | EACH07 | 19 | 6 | 0.61 | 0.74 | 5.29 | 0.175 | 0.462 | -0.103 |
|  | EACH08 | 19 | 4 | 0.50 | 0.62 | 3.74 | 0.192 | 0.534 | -0.140 |
|  | EACH09 | 19 | 10 | 0.94 | 0.83 | 8.06 | -0.142 | -0.025 | -0.249 |
|  | EACH10 | 19 | 4 | 0.39 | 0.33 | 3.32 | -0.161 | -0.055 | -0.290 |
|  | EACH11 | 19 | 6 | 0.72 | 0.78 | 5.48 | 0.071 | 0.306 | -0.151 |
|  | EACH12 | 19 | 7 | 0.72 | 0.72 | 5.94 | 0.002 | 0.240 | -0.232 |
|  | Overall | 19 | 6.00 | 0.72 | 0.70 | 5.23 | -0.024 | 0.043 | -0.099 |
| 833 | |  |  |  |  |  |  |  |  |
|  | EACH01 | 24 | 5 | 0.83 | 0.72 | 4.87 | -0.153 | 0.037 | -0.329 |
|  | EACH02 | 21 | 6 | 0.86 | 0.80 | 5.73 | -0.075 | 0.115 | -0.242 |
|  | EACH03 | 24 | 6 | 0.58 | 0.73 | 4.80 | 0.206 | 0.492 | -0.042 |
|  | EACH04 | 24 | 10 | 0.79 | 0.82 | 7.82 | 0.029 | 0.274 | -0.192 |
|  | EACH05 | 24 | 7 | 0.92 | 0.82 | 6.17 | -0.120 | 0.023 | -0.234 |
|  | EACH06 | 20 | 5 | 0.60 | 0.63 | 4.49 | 0.046 | 0.336 | -0.219 |
|  | EACH07 | 24 | 8 | 0.54 | 0.70 | 6.20 | 0.229 | 0.512 | -0.056 |
|  | EACH08 | 23 | 5 | 0.61 | 0.63 | 4.11 | 0.029 | 0.364 | -0.307 |
|  | EACH09 | 24 | 11 | 0.92 | 0.87 | 9.03 | -0.051 | 0.097 | -0.164 |
|  | EACH10 | 24 | 5 | 0.58 | 0.47 | 4.08 | -0.247 | -0.143 | -0.364 |
|  | EACH11 | 24 | 9 | 0.67 | 0.78 | 7.01 | 0.143 | 0.360 | -0.057 |
|  | EACH12 | 24 | 7 | 0.58 | 0.57 | 5.32 | -0.021 | 0.256 | -0.266 |
|  | Overall | 24 | 7.00 | 0.71 | 0.71 | 5.80 | 0.006 | 0.080 | -0.065 |
| 613 | |  |  |  |  |  |  |  |  |
|  | EACH01 | 24 | 6 | 0.67 | 0.68 | 4.98 | 0.017 | 0.246 | -0.176 |
|  | EACH02 | 25 | 8 | 0.72 | 0.81 | 6.96 | 0.116 | 0.331 | -0.084 |
|  | EACH03 | 26 | 8 | 0.77 | 0.78 | 6.06 | 0.009 | 0.242 | -0.196 |
|  | EACH04 | 24 | 9 | 0.83 | 0.79 | 7.42 | -0.055 | 0.122 | -0.217 |
|  | EACH05 | 25 | 7 | 0.67 | 0.65 | 5.66 | 0.016 | 0.249 | -0.197 |
|  | EACH06 | 24 | 8 | 0.83 | 0.84 | 7.02 | 0.013 | 0.219 | -0.163 |
|  | EACH07 | 24 | 7 | 0.67 | 0.65 | 5.66 | -0.025 | 0.222 | -0.246 |
|  | EACH08 | 24 | 3 | 0.58 | 0.53 | 2.69 | -0.111 | 0.241 | -0.441 |
|  | EACH09 | 24 | 10 | 0.83 | 0.78 | 6.71 | -0.075 | 0.135 | -0.269 |
|  | EACH10 | 24 | 5 | 0.42 | 0.39 | 3.93 | -0.067 | 0.129 | -0.209 |
|  | EACH11 | 24 | 10 | 0.88 | 0.87 | 8.25 | -0.007 | 0.167 | -0.154 |
|  | EACH12 | 24 | 8 | 0.83 | 0.84 | 5.79 | 0.099 | 0.381 | -0.160 |
|  | Overall | 26 | 7.42 | 0.70 | 0.70 | 5.87 | -0.001 | 0.082 | -0.078 |
| 896 | |  |  |  |  |  |  |  |  |
|  | EACH01 | 30 | 6 | 0.73 | 0.76 | 5.26 | 0.032 | 0.240 | -0.151 |
|  | EACH02 | 30 | 5 | 0.63 | 0.65 | 3.77 | 0.025 | 0.277 | -0.196 |
|  | EACH03 | 29 | 4 | 0.62 | 0.65 | 3.97 | 0.047 | 0.291 | -0.170 |
|  | EACH04 | 29 | 7 | 0.86 | 0.81 | 5.98 | -0.063 | 0.091 | -0.202 |
|  | EACH05 | 30 | 6 | 0.67 | 0.73 | 4.69 | 0.083 | 0.298 | -0.136 |
|  | EACH06 | 26 | 10 | 0.96 | 0.85 | 8.42 | -0.134 | -0.051 | -0.214 |
|  | EACH07 | 30 | 7 | 0.63 | 0.80 | 5.89 | 0.204 | 0.419 | 0.002 |
|  | EACH08 | 28 | 4 | 0.46 | 0.67 | 3.34 | 0.306 | 0.580 | 0.018 |
|  | EACH09 | 30 | 11 | 0.80 | 0.79 | 7.63 | -0.010 | 0.186 | -0.190 |
|  | EACH10 | 30 | 6 | 0.37 | 0.32 | 3.89 | -0.136 | -0.069 | -0.227 |
|  | EACH11 | 30 | 8 | 0.77 | 0.85 | 7.47 | 0.097 | 0.284 | -0.080 |
|  | EACH12 | 30 | 8 | 0.73 | 0.80 | 6.47 | 0.088 | 0.276 | -0.099 |
|  | Overall | 30 | 6.83 | 0.69 | 0.72 | 5.57 | 0.050 | 0.095 | 0.008 |
| 561 | |  |  |  |  |  |  |  |  |
|  | EACH01 | 33 | 8 | 0.73 | 0.79 | 5.84 | 0.084 | 0.279 | -0.097 |
|  | EACH02 | 34 | 6 | 0.79 | 0.77 | 5.64 | -0.036 | 0.138 | -0.195 |
|  | EACH03 | 32 | 8 | 0.75 | 0.70 | 6.28 | -0.066 | 0.107 | -0.218 |
|  | EACH04 | 33 | 9 | 0.91 | 0.85 | 8.17 | -0.070 | 0.046 | -0.186 |
|  | EACH05 | 34 | 8 | 0.85 | 0.83 | 6.90 | -0.030 | 0.123 | -0.161 |
|  | EACH06 | 34 | 11 | 0.79 | 0.82 | 8.85 | 0.036 | 0.175 | -0.092 |
|  | EACH07 | 33 | 8 | 0.52 | 0.69 | 5.58 | **0.253** | **0.507** | **0.005** |
|  | EACH08 | 30 | 7 | 0.57 | 0.72 | 5.19 | 0.214 | 0.435 | 0.002 |
|  | EACH09 | 34 | 15 | 0.76 | 0.85 | 9.68 | 0.098 | 0.256 | -0.052 |
|  | EACH10 | 34 | 5 | 0.50 | 0.45 | 3.29 | -0.123 | 0.120 | -0.348 |
|  | EACH11 | 34 | 11 | 0.74 | 0.76 | 6.98 | 0.033 | 0.239 | -0.146 |
|  | EACH12 | 34 | 11 | 0.71 | 0.71 | 6.77 | 0.008 | 0.198 | -0.164 |
|  | Overall | 34 | 8.92 | 0.72 | 0.75 | 6.60 | 0.036 | 0.083 | -0.011 |
| 960 | |  |  |  |  |  |  |  |  |
|  | EACH01 | 17 | 6 | 0.41 | 0.70 | 5.15 | 0.415 | 0.731 | 0.129 |
|  | EACH02 | 17 | 6 | 0.76 | 0.79 | 5.40 | 0.029 | 0.296 | -0.248 |
|  | EACH03 | 17 | 4 | 0.71 | 0.73 | 3.99 | -0.037 | 0.174 | -0.212 |
|  | EACH04 | 17 | 9 | 0.94 | 0.85 | 7.99 | -0.103 | 0.064 | -0.245 |
|  | EACH05 | 17 | 5 | 0.53 | 0.61 | 4.32 | 0.138 | 0.504 | -0.228 |
|  | EACH06 | 17 | 4 | 0.59 | 0.60 | 3.80 | 0.020 | 0.324 | -0.234 |
|  | EACH07 | 16 | 7 | 0.44 | 0.78 | 5.84 | 0.436 | 0.740 | 0.110 |
|  | EACH08 | 17 | 3 | 0.59 | 0.59 | 2.99 | 0.009 | 0.403 | -0.373 |
|  | EACH09 | 17 | 10 | 0.88 | 0.88 | 8.89 | -0.002 | 0.193 | -0.146 |
|  | EACH10 | 17 | 5 | 0.76 | 0.71 | 4.68 | -0.075 | 0.186 | -0.308 |
|  | EACH11 | 17 | 8 | 0.88 | 0.85 | 7.28 | -0.037 | 0.174 | -0.212 |
|  | EACH12 | 17 | 7 | 0.82 | 0.79 | 6.43 | -0.046 | 0.164 | -0.227 |
|  | Overall | 17 | 6.17 | 0.69 | 0.74 | 5.56 | 0.064 | 0.129 | -0.013 |
| 920 | |  |  |  |  |  |  |  |  |
|  | EACH01 | 40 | 6 | 0.70 | 0.68 | 4.68 | -0.032 | 0.155 | -0.200 |
|  | EACH02 | 40 | 7 | 0.60 | 0.71 | 4.95 | 0.156 | 0.353 | -0.035 |
|  | EACH03 | 40 | 6 | 0.72 | 0.79 | 5.56 | 0.082 | 0.269 | -0.095 |
|  | EACH04 | 40 | 7 | 0.80 | 0.75 | 5.96 | -0.061 | 0.101 | -0.204 |
|  | EACH05 | 40 | 3 | 0.82 | 0.73 | 3.98 | -0.133 | 0.040 | -0.293 |
|  | EACH06 | 40 | 11 | 0.70 | 0.86 | 8.46 | 0.181 | 0.349 | 0.028 |
|  | EACH07 | 40 | 10 | 0.82 | 0.85 | 8.06 | 0.032 | 0.168 | -0.091 |
|  | EACH08 | 40 | 3 | 0.70 | 0.62 | 2.99 | -0.124 | 0.099 | -0.336 |
|  | EACH09 | 40 | 14 | 0.90 | 0.90 | 10.58 | 0.003 | 0.120 | -0.090 |
|  | EACH10 | 40 | 6 | 0.48 | 0.56 | 4.52 | 0.155 | 0.385 | -0.059 |
|  | EACH11 | 40 | 10 | 0.85 | 0.78 | 7.47 | -0.084 | 0.033 | -0.206 |
|  | EACH12 | 40 | 11 | 0.78 | 0.80 | 7.87 | 0.028 | 0.171 | -0.115 |
|  | Overall | 40 | 7.92 | 0.74 | 0.75 | 6.26 | 0.018 | 0.063 | -0.033 |
| 793 | |  |  |  |  |  |  |  |  |
|  | EACH01 | 40 | 8 | 0.60 | 0.65 | 6.33 | 0.076 | 0.261 | -0.091 |
|  | EACH02 | 40 | 5 | 0.65 | 0.68 | 4.05 | 0.043 | 0.261 | -0.157 |
|  | EACH03 | 39 | 8 | 0.64 | 0.69 | 5.26 | 0.067 | 0.287 | -0.139 |
|  | EACH04 | 38 | 7 | 0.84 | 0.79 | 6.38 | -0.060 | 0.067 | -0.174 |
|  | EACH05 | 40 | 7 | 0.80 | 0.72 | 5.57 | -0.107 | 0.050 | -0.258 |
|  | EACH06 | 39 | 9 | 0.74 | 0.82 | 6.67 | 0.096 | 0.253 | -0.056 |
|  | EACH07 | 39 | 5 | 0.56 | 0.66 | 4.35 | 0.150 | 0.387 | -0.086 |
|  | EACH08 | 39 | 3 | 0.56 | 0.63 | 3.00 | 0.105 | 0.341 | -0.120 |
|  | EACH09 | 40 | 12 | 0.85 | 0.87 | 9.74 | 0.023 | 0.135 | -0.077 |
|  | EACH10 | 39 | 3 | 0.36 | 0.34 | 2.84 | -0.043 | 0.258 | -0.232 |
|  | EACH11 | 40 | 10 | 0.88 | 0.85 | 8.00 | -0.033 | 0.089 | -0.148 |
|  | EACH12 | 40 | 8 | 0.85 | 0.83 | 6.72 | -0.025 | 0.110 | -0.151 |
|  | Overall | 40 | 7.08 | 0.69 | 0.71 | 5.74 | 0.024 | 0.067 | -0.023 |
| 790 | |  |  |  |  |  |  |  |  |
|  | EACH01 | 41 | 7 | 0.74 | 0.81 | 6.15 | 0.089 | 0.265 | -0.065 |
|  | EACH02 | 41 | 9 | 0.86 | 0.79 | 6.98 | -0.080 | 0.084 | -0.199 |
|  | EACH03 | 41 | 6 | 0.76 | 0.70 | 4.48 | -0.096 | 0.084 | -0.253 |
|  | EACH04 | 40 | 9 | 0.68 | 0.86 | 7.74 | 0.202 | 0.367 | 0.042 |
|  | EACH05 | 41 | 9 | 0.88 | 0.80 | 7.34 | -0.096 | 0.017 | -0.198 |
|  | EACH06 | 40 | 9 | 0.73 | 0.77 | 6.81 | 0.045 | 0.200 | -0.104 |
|  | EACH07 | 40 | 7 | 0.37 | 0.74 | 4.97 | 0.503 | 0.704 | 0.287 |
|  | EACH08 | 39 | 3 | 0.32 | 0.34 | 2.29 | 0.043 | 0.365 | -0.220 |
|  | EACH09 | 41 | 13 | 0.86 | 0.89 | 9.40 | 0.033 | 0.150 | -0.072 |
|  | EACH10 | 41 | 7 | 0.48 | 0.44 | 4.98 | -0.080 | 0.067 | -0.193 |
|  | EACH11 | 41 | 10 | 0.48 | 0.68 | 6.19 | 0.303 | 0.517 | 0.080 |
|  | EACH12 | 41 | 9 | 0.64 | 0.68 | 6.20 | 0.058 | 0.246 | -0.107 |
|  | Overall | 41 | 8.17 | 0.65 | 0.71 | 6.13 | **0.082** | **0.133** | **0.038** |
| 831 | |  |  |  |  |  |  |  |  |
|  | EACH01 | 34 | 6 | 0.69 | 0.73 | 5.00 | 0.058 | 0.287 | -0.154 |
|  | EACH02 | 34 | 6 | 0.62 | 0.76 | 5.37 | 0.181 | 0.408 | -0.041 |
|  | EACH03 | 34 | 5 | 0.65 | 0.58 | 4.10 | -0.111 | 0.106 | -0.287 |
|  | EACH04 | 34 | 7 | 0.81 | 0.82 | 6.17 | 0.019 | 0.206 | -0.139 |
|  | EACH05 | 34 | 7 | 0.84 | 0.74 | 5.64 | -0.138 | 0.032 | -0.287 |
|  | EACH06 | 34 | 9 | 0.75 | 0.75 | 6.58 | -0.005 | 0.157 | -0.144 |
|  | EACH07 | 34 | 11 | 0.59 | 0.82 | 7.93 | 0.277 | 0.466 | 0.088 |
|  | EACH08 | 34 | 3 | 0.56 | 0.56 | 2.99 | 0.001 | 0.218 | -0.215 |
|  | EACH09 | 34 | 10 | 0.88 | 0.86 | 8.20 | -0.018 | 0.122 | -0.148 |
|  | EACH10 | 34 | 5 | 0.38 | 0.37 | 3.81 | -0.005 | 0.191 | -0.156 |
|  | EACH11 | 34 | 11 | 0.84 | 0.88 | 9.32 | 0.046 | 0.187 | -0.086 |
|  | EACH12 | 34 | 7 | 0.78 | 0.77 | 6.01 | -0.014 | 0.163 | -0.189 |
|  | Overall | 34 | 7.25 | 0.70 | 0.72 | 5.93 | 0.031 | 0.093 | -0.035 |
| 845 | |  |  |  |  |  |  |  |  |
|  | EACH01 | 19 | 6 | 0.84 | 0.73 | 5.20 | -0.160 | 0.006 | -0.345 |
|  | EACH02 | 19 | 3 | 0.58 | 0.54 | 2.80 | -0.080 | 0.299 | -0.462 |
|  | EACH03 | 19 | 5 | 0.79 | 0.70 | 4.33 | -0.133 | 0.109 | -0.395 |
|  | EACH04 | 19 | 7 | 0.84 | 0.82 | 6.72 | -0.024 | 0.182 | -0.205 |
|  | EACH05 | 19 | 6 | 0.74 | 0.67 | 4.86 | -0.099 | 0.142 | -0.318 |
|  | EACH06 | 19 | 7 | 0.58 | 0.77 | 5.86 | 0.244 | 0.518 | -0.022 |
|  | EACH07 | 19 | 5 | 0.53 | 0.78 | 4.87 | 0.321 | 0.611 | 0.015 |
|  | EACH08 | 19 | 3 | 0.68 | 0.62 | 3.00 | -0.095 | 0.239 | -0.434 |
|  | EACH09 | 19 | 8 | 0.74 | 0.78 | 6.56 | 0.057 | 0.291 | -0.131 |
|  | EACH10 | 19 | 4 | 0.42 | 0.43 | 3.54 | 0.023 | 0.428 | -0.283 |
|  | EACH11 | 19 | 7 | 0.74 | 0.79 | 6.27 | 0.068 | 0.307 | -0.143 |
|  | EACH12 | 19 | 5 | 0.63 | 0.65 | 4.30 | 0.022 | 0.299 | -0.233 |
|  | Overall | 19 | 5.50 | 0.68 | 0.69 | 4.86 | 0.019 | 0.076 | -0.044 |
| 854 | |  |  |  |  |  |  |  |  |
|  | EACH01 | 54 | 7 | 0.63 | 0.64 | 5.14 | 0.023 | 0.187 | -0.126 |
|  | EACH02 | 56 | 6 | 0.61 | 0.69 | 4.09 | 0.121 | 0.303 | -0.044 |
|  | EACH03 | 54 | 9 | 0.65 | 0.79 | 6.34 | 0.175 | 0.345 | 0.018 |
|  | EACH04 | 55 | 10 | 0.82 | 0.80 | 7.13 | -0.019 | 0.107 | -0.136 |
|  | EACH05 | 56 | 11 | 0.66 | 0.74 | 5.88 | 0.110 | 0.251 | -0.033 |
|  | EACH06 | 54 | 12 | 0.80 | 0.83 | 8.17 | 0.038 | 0.155 | -0.069 |
|  | EACH07 | 51 | 11 | 0.41 | 0.80 | 6.79 | **0.485** | **0.634** | **0.334** |
|  | EACH08 | 51 | 3 | 0.61 | 0.54 | 2.93 | -0.130 | 0.084 | -0.327 |
|  | EACH09 | 55 | 15 | 0.91 | 0.89 | 10.07 | 0.066 | 0.175 | -0.037 |
|  | EACH10 | 54 | 7 | 0.67 | 0.63 | 4.96 | -0.058 | 0.099 | -0.213 |
|  | EACH11 | 56 | 14 | 0.82 | 0.88 | 10.12 | 0.066 | 0.175 | -0.037 |
|  | EACH12 | 54 | 10 | 0.76 | 0.78 | 7.48 | 0.029 | 0.142 | -0.078 |
|  | Overall | 56 | 9.58 | 0.69 | 0.75 | 6.59 | **0.075** | **0.114** | **0.034** |
| 856 | |  |  |  |  |  |  |  |  |
|  | EACH01 | 18 | 6 | 0.61 | 0.71 | 5.17 | 0.143 | 0.492 | -0.203 |
|  | EACH02 | 18 | 5 | 0.89 | 0.73 | 4.86 | -0.213 | 0.060 | -0.457 |
|  | EACH03 | 18 | 5 | 0.61 | 0.70 | 4.71 | 0.132 | 0.427 | -0.154 |
|  | EACH04 | 18 | 9 | 1.00 | 0.84 | 7.98 | -0.196 | -0.133 | -0.301 |
|  | EACH05 | 18 | 6 | 0.94 | 0.76 | 5.46 | -0.246 | -0.112 | -0.387 |
|  | EACH06 | 18 | 6 | 0.72 | 0.64 | 5.47 | -0.122 | 0.083 | -0.330 |
|  | EACH07 | 18 | 7 | 0.72 | 0.72 | 5.55 | 0.000 | 0.321 | -0.292 |
|  | EACH08 | 18 | 3 | 0.78 | 0.59 | 2.99 | -0.316 | -0.018 | -0.576 |
|  | EACH09 | 18 | 11 | 0.94 | 0.86 | 8.96 | -0.097 | 0.047 | -0.221 |
|  | EACH10 | 18 | 5 | 0.28 | 0.30 | 3.73 | 0.067 | 0.322 | -0.123 |
|  | EACH11 | 18 | 9 | 0.89 | 0.83 | 8.04 | -0.065 | 0.100 | -0.208 |
|  | EACH12 | 18 | 6 | 0.50 | 0.42 | 4.40 | -0.191 | -0.085 | -0.318 |
|  | Overall | 18 | 6.50 | 0.74 | 0.68 | 5.61 | -0.096 | -0.036 | -0.164 |
| 869 | |  |  |  |  |  |  |  |  |
|  | EACH01 | 32 | 6 | 0.76 | 0.79 | 5.14 | 0.036 | 0.232 | -0.158 |
|  | EACH02 | 32 | 4 | 0.52 | 0.58 | 3.37 | 0.110 | 0.377 | -0.157 |
|  | EACH03 | 32 | 6 | 0.85 | 0.76 | 5.30 | -0.117 | 0.038 | -0.279 |
|  | EACH04 | 32 | 6 | 0.76 | 0.82 | 5.88 | 0.075 | 0.268 | -0.095 |
|  | EACH05 | 32 | 6 | 0.67 | 0.75 | 5.18 | 0.111 | 0.309 | -0.091 |
|  | EACH06 | 32 | 10 | 1.00 | 0.87 | 8.69 | -0.146 | -0.119 | -0.187 |
|  | EACH07 | 32 | 8 | 0.64 | 0.70 | 6.11 | 0.086 | 0.284 | -0.096 |
|  | EACH08 | 32 | 5 | 0.73 | 0.71 | 4.54 | -0.023 | 0.167 | -0.218 |
|  | EACH09 | 32 | 10 | 0.76 | 0.83 | 8.14 | 0.092 | 0.262 | -0.059 |
|  | EACH10 | 32 | 7 | 0.55 | 0.49 | 4.70 | -0.120 | 0.033 | -0.272 |
|  | EACH11 | 32 | 9 | 0.85 | 0.83 | 7.33 | -0.019 | 0.142 | -0.168 |
|  | EACH12 | 32 | 10 | 0.79 | 0.79 | 7.08 | 0.001 | 0.184 | -0.167 |
|  | Overall | 32 | 7.25 | 0.74 | 0.74 | 5.96 | 0.008 | 0.062 | -0.051 |
| 844 | |  |  |  |  |  |  |  |  |
|  | EACH01 | 53 | 5 | 0.74 | 0.74 | 4.24 | 0.009 | 0.168 | -0.156 |
|  | EACH02 | 52 | 7 | 0.29 | 0.51 | 4.32 | 0.440 | 0.653 | 0.217 |
|  | EACH03 | 53 | 6 | 0.75 | 0.73 | 5.08 | -0.030 | 0.110 | -0.158 |
|  | EACH04 | 53 | 8 | 0.66 | 0.78 | 6.57 | 0.151 | 0.286 | 0.025 |
|  | EACH05 | 53 | 7 | 0.74 | 0.78 | 5.75 | 0.054 | 0.199 | -0.085 |
|  | EACH06 | 51 | 11 | 0.86 | 0.87 | 8.66 | 0.003 | 0.112 | -0.094 |
|  | EACH07 | 53 | 8 | 0.57 | 0.66 | 6.12 | 0.138 | 0.283 | -0.006 |
|  | EACH08 | 52 | 4 | 0.58 | 0.70 | 3.94 | 0.172 | 0.365 | -0.027 |
|  | EACH09 | 53 | 11 | 0.89 | 0.86 | 8.70 | -0.017 | 0.090 | -0.109 |
|  | EACH10 | 53 | 6 | 0.32 | 0.29 | 3.57 | -0.118 | -0.062 | -0.174 |
|  | EACH11 | 53 | 11 | 0.89 | 0.86 | 8.17 | -0.034 | 0.065 | -0.129 |
|  | EACH12 | 53 | 8 | 0.75 | 0.83 | 6.86 | 0.085 | 0.243 | -0.046 |
|  | Overall | 53 | 7.83 | 0.66 | 0.71 | 6.00 | 0.068 | 0.108 | 0.029 |
| 905 | |  |  |  |  |  |  |  |  |
|  | EACH01 | 31 | 5 | 0.69 | 0.74 | 4.55 | 0.076 | 0.283 | -0.115 |
|  | EACH02 | 31 | 4 | 0.41 | 0.53 | 3.55 | 0.236 | 0.525 | -0.044 |
|  | EACH03 | 31 | 7 | 0.84 | 0.82 | 6.34 | -0.031 | 0.131 | -0.185 |
|  | EACH04 | 30 | 9 | 0.84 | 0.86 | 7.92 | 0.030 | 0.190 | -0.111 |
|  | EACH05 | 31 | 8 | 0.72 | 0.72 | 5.81 | 0.002 | 0.208 | -0.193 |
|  | EACH06 | 31 | 8 | 0.56 | 0.66 | 5.89 | 0.149 | 0.364 | -0.042 |
|  | EACH07 | 30 | 7 | 0.61 | 0.76 | 5.22 | 0.196 | 0.421 | -0.024 |
|  | EACH08 | 31 | 3 | 0.44 | 0.44 | 2.97 | 0.014 | 0.266 | -0.208 |
|  | EACH09 | 31 | 14 | 0.91 | 0.90 | 10.67 | -0.011 | 0.102 | -0.109 |
|  | EACH10 | 31 | 6 | 0.66 | 0.55 | 4.16 | -0.200 | -0.010 | -0.387 |
|  | EACH11 | 31 | 10 | 0.97 | 0.86 | 8.53 | -0.132 | -0.051 | -0.198 |
|  | EACH12 | 31 | 5 | 0.66 | 0.62 | 4.61 | -0.061 | 0.090 | -0.221 |
|  | Overall | 31 | 7.17 | 0.69 | 0.71 | 5.85 | 0.020 | 0.067 | -0.026 |
| 870 | |  |  |  |  |  |  |  |  |
|  | EACH01 | 29 | 8 | 0.69 | 0.79 | 6.83 | 0.124 | 0.305 | -0.045 |
|  | EACH02 | 30 | 6 | 0.80 | 0.73 | 4.86 | -0.090 | 0.101 | -0.272 |
|  | EACH03 | 29 | 6 | 0.83 | 0.76 | 5.24 | -0.092 | 0.060 | -0.245 |
|  | EACH04 | 29 | 9 | 0.79 | 0.85 | 7.27 | 0.063 | 0.233 | -0.084 |
|  | EACH05 | 29 | 9 | 0.90 | 0.82 | 7.15 | -0.090 | 0.050 | -0.215 |
|  | EACH06 | 29 | 9 | 0.76 | 0.66 | 5.66 | -0.150 | 0.027 | -0.327 |
|  | EACH07 | 29 | 9 | 0.48 | 0.60 | 5.60 | 0.194 | 0.447 | -0.055 |
|  | EACH08 | 27 | 4 | 0.41 | 0.52 | 2.98 | 0.222 | 0.546 | -0.114 |
|  | EACH09 | 29 | 14 | 0.90 | 0.91 | 10.61 | 0.011 | 0.150 | -0.113 |
|  | EACH10 | 29 | 7 | 0.66 | 0.64 | 5.57 | -0.030 | 0.205 | -0.250 |
|  | EACH11 | 29 | 10 | 0.93 | 0.84 | 7.56 | -0.107 | 0.032 | -0.222 |
|  | EACH12 | 29 | 6 | 0.62 | 0.60 | 4.63 | -0.031 | 0.189 | -0.222 |
|  | Overall | 30 | 8.08 | 0.73 | 0.73 | 6.16 | -0.005 | 0.052 | -0.058 |
| 826 | |  |  |  |  |  |  |  |  |
|  | EACH01 | 30 | 10 | 0.79 | 0.83 | 7.11 | 0.041 | 0.213 | -0.120 |
|  | EACH02 | 31 | 5 | 0.63 | 0.64 | 4.29 | 0.011 | 0.271 | -0.240 |
|  | EACH03 | 31 | 6 | 0.80 | 0.76 | 5.12 | -0.056 | 0.122 | -0.227 |
|  | EACH04 | 30 | 9 | 0.83 | 0.81 | 7.37 | -0.021 | 0.131 | -0.159 |
|  | EACH05 | 31 | 8 | 0.67 | 0.74 | 6.34 | 0.093 | 0.301 | -0.100 |
|  | EACH06 | 29 | 11 | 0.57 | 0.72 | 6.80 | 0.209 | 0.438 | 0.000 |
|  | EACH07 | 30 | 9 | 0.72 | 0.75 | 6.50 | 0.029 | 0.238 | -0.174 |
|  | EACH08 | 30 | 6 | 0.76 | 0.76 | 5.11 | 0.003 | 0.193 | -0170 |
|  | EACH09 | 30 | 14 | 0.86 | 0.89 | 9.92 | 0.034 | 0.184 | -0.102 |
|  | EACH10 | 30 | 5 | 0.55 | 0.49 | 3.91 | -0.136 | 0.047 | -0.293 |
|  | EACH11 | 30 | 9 | 0.59 | 0.75 | 5.96 | 0.221 | 0.457 | -0.010 |
|  | EACH12 | 30 | 8 | 0.90 | 0.80 | 6.57 | -0.117 | -0.001 | -0.216 |
|  | Overall | 31 | 8.33 | 0.72 | 0.74 | 6.25 | 0.027 | 0.086 | -0.027 |
| 803 | |  |  |  |  |  |  |  |  |
|  | EACH01 | 30 | 7 | 0.70 | 0.78 | 5.80 | 0.970 | 0.311 | -0.094 |
|  | EACH02 | 30 | 9 | 0.47 | 0.71 | 6.56 | 0.342 | 0.560 | 0.106 |
|  | EACH03 | 30 | 8 | 0.90 | 0.77 | 6.18 | -0.173 | -0.010 | -0.329 |
|  | EACH04 | 30 | 12 | 0.83 | 0.87 | 8.94 | 0.046 | 0.199 | -0.106 |
|  | EACH05 | 30 | 9 | 0.73 | 0.84 | 7.30 | 0.124 | 0.327 | -0.082 |
|  | EACH06 | 30 | 7 | 0.67 | 0.72 | 5.17 | 0.068 | 0.283 | -0.128 |
|  | EACH07 | 30 | 4 | 0.67 | 0.57 | 3.62 | -0.177 | 0.044 | -0.370 |
|  | EACH08 | 30 | 6 | 0.63 | 0.58 | 4.73 | -0.088 | 0.086 | -0.283 |
|  | EACH09 | 30 | 11 | 0.87 | 0.81 | 7.86 | -0.071 | 0.063 | -0.201 |
|  | EACH10 | 30 | 6 | 0.93 | 0.75 | 5.56 | -0.251 | -0.089 | -0.393 |
|  | EACH11 | 30 | 13 | 0.97 | 0.89 | 9.99 | -0.083 | 0.015 | -0.153 |
|  | EACH12 | 30 | 7 | 0.60 | 0.58 | 5.27 | -0.032 | 0.122 | -0.203 |
|  | Overall | 30 | 8.25 | 0.75 | 0.74 | 6.42 | -0.013 | 0.044 | -0.072 |
| 898 | |  |  |  |  |  |  |  |  |
|  | EACH01 | 23 | 7 | 0.87 | 0.84 | 6.75 | -0.035 | 0.144 | -0.195 |
|  | EACH02 | 23 | 7 | 0.91 | 0.79 | 5.89 | -0.160 | 0.001 | -0.294 |
|  | EACH03 | 23 | 4 | 0.78 | 0.69 | 3.99 | -0.133 | 0.099 | -0.326 |
|  | EACH04 | 23 | 7 | 0.78 | 0.82 | 6.33 | 0.043 | 0.239 | -0.150 |
|  | EACH05 | 23 | 7 | 0.61 | 0.75 | 5.27 | 0.191 | 0.445 | -0.068 |
|  | EACH06 | 23 | 7 | 0.57 | 0.65 | 5.13 | 0.131 | 0.358 | -0.079 |
|  | EACH07 | 23 | 9 | 0.74 | 0.81 | 7.30 | 0.085 | 0.331 | -0.132 |
|  | EACH08 | 23 | 4 | 0.48 | 0.51 | 2.94 | 0.058 | 0.410 | -0.271 |
|  | EACH09 | 23 | 10 | 0.83 | 0.83 | 8.46 | 0.002 | 0.166 | -0.147 |
|  | EACH10 | 23 | 6 | 0.61 | 0.54 | 4.92 | -0.120 | 0.056 | -0.290 |
|  | EACH11 | 23 | 10 | 0.78 | 0.79 | 7.94 | 0.004 | 0.210 | -0.197 |
|  | EACH12 | 23 | 8 | 0.61 | 0.62 | 6.11 | 0.023 | 0.190 | -0.151 |
|  | Overall | 23 | 7.17 | 0.71 | 0.72 | 5.92 | 0.008 | 0.051 | -0.042 |
| 875 | |  |  |  |  |  |  |  |  |
|  | EACH01 | 26 | 7 | 0.88 | 0.83 | 6.59 | -0.065 | 0.083 | -0.210 |
|  | EACH02 | 26 | 6 | 0.69 | 0.74 | 4.96 | 0.071 | 0.329 | -0.186 |
|  | EACH03 | 25 | 6 | 0.72 | 0.63 | 4.83 | -0.139 | 0.098 | -0.343 |
|  | EACH04 | 25 | 9 | 0.76 | 0.81 | 6.68 | 0.061 | 0.280 | -0.134 |
|  | EACH05 | 26 | 8 | 0.92 | 0.85 | 7.64 | -0.082 | 0.048 | -0.191 |
|  | EACH06 | 26 | 7 | 0.62 | 0.59 | 5.51 | -0.052 | 0.198 | -0.241 |
|  | EACH07 | 26 | 8 | 0.73 | 0.72 | 6.04 | -0.008 | 0.224 | -0.250 |
|  | EACH08 | 26 | 2 | 0.54 | 0.49 | 2.00 | -0.103 | 0.304 | -0.495 |
|  | EACH09 | 25 | 8 | 0.80 | 0.70 | 6.37 | -0.136 | 0.025 | -0.313 |
|  | EACH10 | 25 | 6 | 0.48 | 0.60 | 4.93 | 0.195 | 0.408 | -0.012 |
|  | EACH11 | 25 | 6 | 0.80 | 0.71 | 5.15 | -0.122 | 0.096 | -0.334 |
|  | EACH12 | 26 | 5 | 0.58 | 0.63 | 4.28 | 0.088 | 0.336 | -0.137 |
|  | Overall | 26 | 6.50 | 0.71 | 0.69 | 5.42 | -0.025 | 0.031 | -0.083 |
| 763 | |  |  |  |  |  |  |  |  |
|  | EACH01 | 35 | 6 | 0.51 | 0.57 | 4.71 | 0.094 | 0.346 | -0.145 |
|  | EACH02 | 35 | 7 | 0.77 | 0.79 | 6.00 | 0.021 | 0.190 | -0.133 |
|  | EACH03 | 35 | 8 | 0.51 | 0.59 | 5.30 | 0.124 | 0.359 | -0.099 |
|  | EACH04 | 35 | 5 | 0.74 | 0.71 | 4.79 | -0.039 | 0.101 | -0.176 |
|  | EACH05 | 35 | 9 | 0.74 | 0.74 | 5.96 | -0.007 | 0.137 | -0.152 |
|  | EACH06 | 35 | 6 | 0.86 | 0.75 | 5.17 | -0.146 | 0.002 | -0.283 |
|  | EACH07 | 35 | 7 | 0.60 | 0.71 | 4.95 | 0.151 | 0.363 | -0.055 |
|  | EACH08 | 35 | 4 | 0.51 | 0.64 | 3.81 | 0.201 | 0.441 | -0.043 |
|  | EACH09 | 35 | 11 | 0.80 | 0.82 | 7.63 | 0.021 | 0.163 | -0.116 |
|  | EACH10 | 35 | 7 | 0.54 | 0.64 | 5.73 | 0.152 | 0.342 | -0.034 |
|  | EACH11 | 35 | 9 | 0.71 | 0.74 | 6.27 | 0.034 | 0.221 | -0.139 |
|  | EACH12 | 35 | 7 | 0.91 | 0.83 | 6.67 | -0.107 | 0.016 | -0.212 |
|  | Overall | 35 | 7.17 | 0.69 | 0.71 | 5.58 | 0.034 | 0.079 | -0.015 |
| 691 | |  |  |  |  |  |  |  |  |
|  | EACH01 | 19 | 6 | 0.74 | 0.69 | 5.07 | -0.066 | 0.216 | -0.309 |
|  | EACH02 | 19 | 6 | 0.79 | 0.72 | 4.99 | -0.090 | 0.144 | -0.291 |
|  | EACH03 | 19 | 5 | 0.42 | 0.44 | 4.37 | 0.041 | 0.242 | -0.179 |
|  | EACH04 | 19 | 7 | 0.79 | 0.83 | 6.66 | 0.053 | 0.253 | -0.141 |
|  | EACH05 | 19 | 5 | 0.63 | 0.57 | 4.06 | -0.101 | 0.111 | -0.314 |
|  | EACH06 | 19 | 9 | 0.84 | 0.84 | 7.45 | -0.005 | 0.197 | -0.181 |
|  | EACH07 | 19 | 7 | 0.63 | 0.66 | 5.44 | 0.042 | 0.339 | -0.231 |
|  | EACH08 | 19 | 3 | 0.53 | 0.64 | 3.00 | 0.183 | 0.523 | -0.175 |
|  | EACH09 | 19 | 8 | 0.84 | 0.79 | 6.69 | -0.065 | 0.145 | -0.242 |
|  | EACH10 | 19 | 3 | 0.53 | 0.44 | 2.93 | -0.184 | 0.028 | -0.398 |
|  | EACH11 | 19 | 4 | 0.58 | 0.67 | 3.80 | 0.138 | 0.429 | -0.153 |
|  | EACH12 | 19 | 7 | 0.79 | 0.72 | 6.33 | -0.094 | 0.142 | -0.295 |
|  | Overall | 19 | 5.83 | 0.68 | 0.67 | 5.07 | -0.009 | 0.068 | -0.086 |
