# Supplementary material for: Assessing the Permeability of Landscape Features to Animal Movement: Using Genetic Structure to Infer Functional Connectivity
Source: PLoS One. 2015 Feb 26;10(2):e0117500. doi: 10.1371/journal.pone.0117500 (PMC4342345; doi:10.1371/journal.pone.0117500)
Supplement: S3 Table — Global FST values were calculated for each study cell as well as pair-wise FST values between all putative clusters within a study cell. All but one FST between putative clusters were significantly different from zero. (DOCX) [file pone.0117500.s003.docx]

**Table S3: Number of genetic clusters estimated from Geneland (*k*) and F_ST_ for each study cell.**

Global F_ST_ values were calculated for each study cell as well as pair-wise F_ST_ values between all putative clusters within a study cell. All but one F_ST_ between putative clusters were significantly different from zero.

| Study Cell | n | k | Global Fst | Significant Fst |
| --- | --- | --- | --- | --- |
| 295 | 144 | 4 | 0.053 | 6/6 |
| 365 | 31 | 2 | 0.042 | 1/1 |
| 366 | 135 | 3 | 0.020 | 3/3 |
| 400 | 28 | 2 | 0.020 | 1/1 |
| 456 | 53 | 2 | 0.054 | 1/1 |
| 580 | 52 | 3 | 0.106 | 3/3 |
| 654 | 67 | 4 | 0.039 | 6/6 |
| 459 | 14 | 1 | Na | Na |
| 464 | 163 | 3 | 0.023 | 3/3 |
| 865 | 27 | 3 | 0.039 | 3/3 |
| 831 | 34 | 1 | Na | Na |
| 833 | 24 | 2 | 0.034 | 1/1 |
| 613 | 26 | 2 | 0.053 | 1/1 |
| 896 | 30 | 2 | 0.045 | 1/1 |
| 561 | 34 | 2 | 0.053 | 1/1 |
| 960 | 17 | 1 | Na | Na |
| 920 | 41 | 2 | 0.018 | 1/1 |
| 793 | 40 | 2 | 0.060 | 1/1 |
| 790 | 41 | 3 | 0.037 | 3/3 |
| 831 | 34 | 3 | 0.057 | 3/3 |
| 845 | 19 | 1 | Na | Na |
| 854 | 56 | 2 | 0.059 | 1/1 |
| 856 | 18 | 1 | Na | Na |
| 869 | 32 | 1 | Na | Na |
| 844 | 53 | 2 | 0.023 | 1/1 |
| 905 | 31 | 1 | Na | Na |
| 870 | 30 | 3 | 0.028 | 3/3 |
| 826 | 32 | 2 | 0.060 | 1/1 |
| 803 | 31 | 3 | 0.037 | 3/3 |
| 898 | 23 | 1 | Na | Na |
| 875 | 26 | 2 | 0.064 | 1/1 |
| 763 | 35 | 2 | 0.075 | 1/1 |
| 691 | 19 | 2 | 0.046 | 1/1 |
