# Supplementary figures and images for: Assessing the Permeability of Landscape Features to Animal Movement: Using Genetic Structure to Infer Functional Connectivity
Source: PLoS One. 2015 Feb 26;10(2):e0117500. doi: 10.1371/journal.pone.0117500 (PMC4342345; doi:10.1371/journal.pone.0117500)

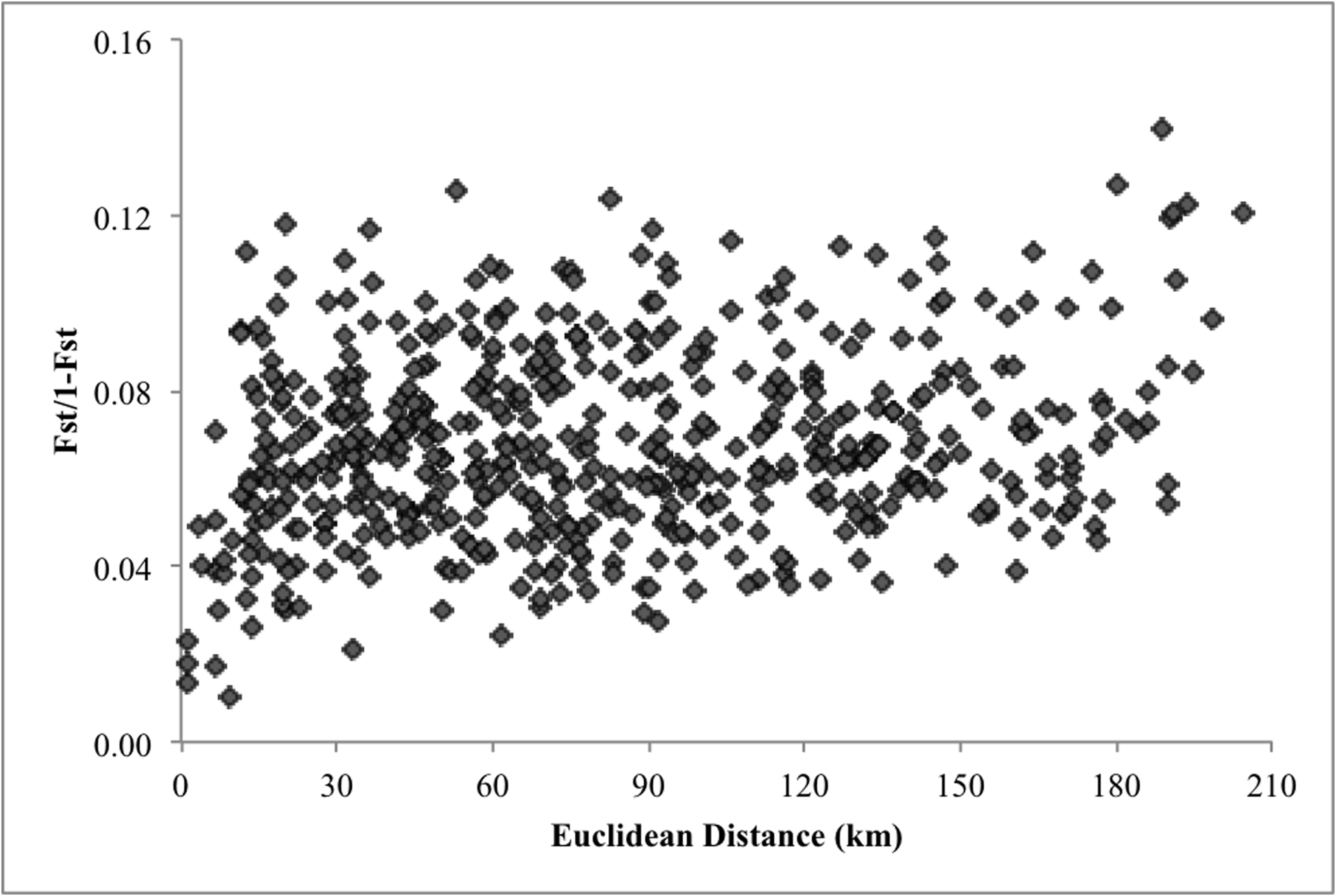

Supplement: S1 Fig — We recorded a positive relationship between geographic and genetic distance, consistent with IBD (r = 0.208, p = 0.003). (TIF) [file pone.0117500.s004.tif]
